# Material surface conjugated with fibroblast growth factor-2 for pluripotent stem cell culture and differentiation

**DOI:** 10.1093/rb/rbaf003

**Published:** 2025-01-02

**Authors:** Tzu-Cheng Sung, Zhi-Xian Pan, Ting Wang, Hui-Yu Lin, Chia-Lun Chang, Ling-Chun Hung, Suresh Kumar Subbiah, Remya Rajan Renuka, Shih-Jie Chou, Shih-Hwa Chiou, Idaszek Joanna, Henry Hsin-Chung Lee, Gwo-Jang Wu, Akon Higuchi

**Affiliations:** State Key Laboratory of Ophthalmology, Optometry and Visual Science, Eye Hospital, Wenzhou Medical University, Wenzhou, Zhejiang 325027, China; Department of Chemical and Materials Engineering, National Central University, Taoyuan 32001, Taiwan, China; State Key Laboratory of Ophthalmology, Optometry and Visual Science, Eye Hospital, Wenzhou Medical University, Wenzhou, Zhejiang 325027, China; Department of Chemical and Materials Engineering, National Central University, Taoyuan 32001, Taiwan, China; Department of Chemical and Materials Engineering, National Central University, Taoyuan 32001, Taiwan, China; Department of Chemical and Materials Engineering, National Central University, Taoyuan 32001, Taiwan, China; Centre for Stem Cell Mediated Advanced Research Therapeutics, Saveetha Dental College and Hospitals, Saveetha Institute of Medical and Technical Sciences, Saveetha University, Chennai 600 077, Tamil Nadu, India; Centre for Stem Cell Mediated Advanced Research Therapeutics, Saveetha Dental College and Hospitals, Saveetha Institute of Medical and Technical Sciences, Saveetha University, Chennai 600 077, Tamil Nadu, India; Department of Medical Research, Taipei Veterans General Hospital, Taipei 112201, Taiwan, China; Institute of Pharmacology, School of Medicine, National Yang Ming Chiao Tung University, Taipei 112304, Taiwan, China; Department of Medical Research, Taipei Veterans General Hospital, Taipei 112201, Taiwan, China; Institute of Pharmacology, School of Medicine, National Yang Ming Chiao Tung University, Taipei 112304, Taiwan, China; Department of Ophthalmology, Taipei Veterans General Hospital, Taipei 11217, Taiwan, China; Division of Materials Design, Faculty of Materials Science and Engineering, Warsaw University of Technology, Warsaw 02-507, Poland; Department of Surgery, Hsinchu Cathay General Hospital, Hsinchu 30060, Taiwan, China; Graduate Institute of Translational and Interdisciplinary Medicine, National Central University, Taoyuan 32001, Taiwan, China; Graduate Institute of Medical Sciences and Department of Obstetrics & Gynecology, Tri-Service General Hospital, National Defense Medical Center, Taipei 11490, Taiwan, China; State Key Laboratory of Ophthalmology, Optometry and Visual Science, Eye Hospital, Wenzhou Medical University, Wenzhou, Zhejiang 325027, China; Department of Chemical and Materials Engineering, National Central University, Taoyuan 32001, Taiwan, China; R&D Center for Membrane Technology, Chung Yuan Christian University, Taoyuan 320, Taiwan, China

**Keywords:** human pluripotent stem cells, carboxymethyl cellulose, fibroblast growth factor-2, cell differentiation, material design

## Abstract

Fibroblast growth factor-2 (FGF-2) is a critical molecule for sustaining the pluripotency of human pluripotent stem (PS) cells. However, FGF-2 is extremely unstable and cannot be stored long periods at room temperature. Therefore, the following FGF-2-conjugated cell culture materials were developed to stabilize FGF-2: FGF-2-conjugated polyvinyl alcohol (PVAI-C-FGF) hydrogels and FGF-2-conjugated carboxymethyl cellulose-coated (CMC-C-FGF) dishes. Human induced pluripotent stem (iPS) cells were proliferated on recombinant vitronectin (rVN)-coated PVAI-C-FGF hydrogels and CMC-C-FGF dishes in medium without FGF-2. Human iPS cells could not be cultivated on rVN-coated PVAI-C-FGF hydrogels for more than two passages but could proliferate on rVN-coated CMC-C-FGF dishes. These results indicated that the amount of immobilized FGF-2 and the base cell materials are important, including the amount of immobilized rVN and the conformation of FGF-2 on the surfaces. When human iPS cells were proliferated on rVN-coated CMC-C-FGF surfaces in medium containing no FGF-2 for 10 passages, their pluripotency and potential to differentiate into cells originating from three germ layers were maintained *in vivo* and *in vitro*. Furthermore, the cells could extensively differentiate into cardiomyocytes, which can be used for cardiac infarction treatment in future and retinal pigment epithelium for retinal pigmentosa treatment in future. The FGF-2-immobilized surface could enable human PS cell culture in medium that does not need to contain unstable FGF-2. The amount of FGF-2 immobilization on the rVN-coated CMC-C-5FGF and CMC-C-20FGF dishes was reduced to 93.6 and 52.2 times, respectively, which is less than the conventional amount of FGF-2 used in culture medium for one passage (6 days) of human iPS cell culture. This reduction resulted from the stabilization of unstable FGF-2 by the immobilization of FGF-2, which was achieved by utilizing optimal base materials (CMC), coating materials (rVN) and long-joint segment (PEG4-SPDP) design.

## Graphical Abstract

**Graphical Abstract:**
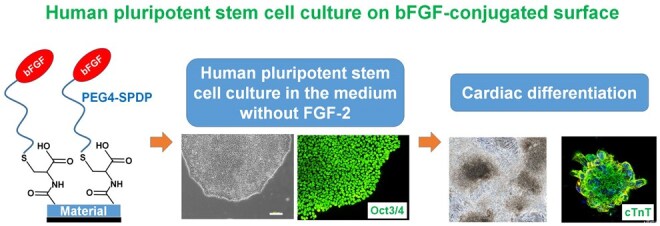

## Introduction

Stem cells hold excellent promise in tissue engineering and regenerative medicine [1–3]. Especially, human pluripotent stem (PS) cells, which are categorized as human embryonic stem (ES) cells or induced pluripotent stem (iPS) cells, could be induced to differentiate into any type of body cells derived from any of the three germ layers. Therefore, cells differentiated from human PS cells are expected to be utilized as cell sources in tissue engineering and regenerative medicine [4–13]. However, human PS cells must be cultured on specific cell culture materials in specific media. Fetal bovine serum (FBS), which is typically a main component of cell culture medium for the cultivation of non-PS cells such as adult stem cells, cancer cells and primary tissue cells, cannot be used to proliferate human PS cells; instead, knockout serum replacement or several combinations of growth factors are utilized to maintain human PS pluripotency. Human PS cells must be cultured in xeno-free or chemically defined media for clinical use. Typically, the chemically defined and xeno-free proliferation medium for human PS cells, Essential 8 (E8) medium, is used for human PS cell cultivation, which is composed of eight substantial components: FGF-2 (fibroblast growth factor-2), TGF (transforming growth factor)-β1, insulin, Dulbecco's modified Eagle's media (DMEM)/F12, sodium selenium, l-ascorbic acid-2-phosphate magnesium, NaHCO_3_ and transferrin [14]. One of the growth factors in E8 medium, FGF-2, is essential to hold the pluripotent state of human PS cells in the cell culture medium, whereas leucocyte inhibition factor (LIF) is critical to maintain mouse ES and iPS cells [15]. However, FGF-2 is an extremely unstable growth factor and should be stored at −20°C for the long-term, such as for more than a few months. The FGF-2 solution is only stable for approximately 7 days at 4°C, and it is recommended to be utilized within one day at room temperature (<25°C) [16, 17]. FGF-2 solution loses 50% of its bioactivity after less than one hour at 25°C [16, 17]. Therefore, several methods have been developed to stabilize FGF-2: (1) genetic engineering [18, 19], (2) physical immobilization with materials or biomolecules [18, 20–26] and (3) chemical immobilization on materials or biomolecules [16, 27–31]. In genetic engineering methods, 1–10 amino acids of FGF-2 are genetically modified to enhance the stability of FGF-2 [18, 19]. However, genetically modified FGF-2 is still unstable and cannot be storaged at room temperature for long time. For the physical immobilization of FGF-2 on materials or biomolecules, heparin, other polysaccharide polymers and negatively charged polymers are added to FGF-2 solution to generate physical complexes between FGF-2 and these biomacromolecules [20–26]. However, FGF-2 is easily released from the materials or biomolecules, the stabilization of FGF-2 is limited. The entrapment of FGF-2 within hydrogels or polymeric microspheres is also reported as a means of physical stabilization of FGF-2 [32, 33]. For the chemical immobilization of FGF-2 on materials or biomolecules, FGF-2 is chemically conjugated to several biomacromolecules and materials, mostly for bone tissue regeneration [16, 27–31].

Nguyen *et al.* conjugated FGF-2 to a heparin-mimicking copolymer, which consisted of a styrene sulfonate group and methyl methacrylate group with poly(ethylene glycol), PEG, and side segments [27], whereas most other researchers conjugated PEG to FGF-2 [34–38]. FGF-2 conjugated to a heparin-mimicking copolymer is expected to be released by the gradual breakage of disulfide bonds between FGF-2 and the copolymer, which is a form of somewhat controlled release [27]. The results of this work indicate that the strategy of conjugating heparin-mimicking copolymers to FGF-2 will be useful to stabilize FGF-2 for future clinical application or research. However, FGF-2 conjugated copolymer has not been utilized for human PS cell culture materials.

Kang *et al.* prepared FGF-2 conjugated PEG by first preparing PEG-maleimide, which reacted with the thiol group of FGF-2 (Michael reaction) [28]. They found that PEGylated FGF-2 showed enhanced penetration into injured spinal cord tissue (T1) in rats from an intrathecal delivery system [28]. The PEGylation of FGF-2 promoted tissue penetration by reducing its rate of elimination. FGF-2 was gradually released from FGF-2-conjugated PEG in this study, with complete release within 24 h [28]. However, the researchers did not consider using FGF-2-conjugated PEG for human PS cell culture materials.

Several studies have suggested that the conjugation of growth factors such as TGF-β2, EGF and FGF-2 to polymers or biomacromolecules does not inhibit the interaction between the growth factors and cell receptor binding sites, making it possible to regulate the localized concentration of growth factors [30, 39].

In previous studies [18, 23–39], FGF-2-conjugated biomolecules or biomaterials have been mainly targeted for tissue engineering, such as bone tissue regeneration and wound healing. Considering the various purposes of FGF-2-immobilized biomaterials, we hypothesized that it might be possible to culture human PS cells on FGF-2-conjugated dishes in chemically defined and xeno-free proliferation media containing no unstable FGF-2. Medium not containing FGF-2 should be more easily stored for a long time under mild conditions such as 4°C. In future, we are considering to develop FGF-2, TGF-β and insulin-conjugated cell culture materials, which can be stored at 4°C for a long time, where human PS cells can be cultured in the medium without growth factors, which can be stored in 4°C. This study is the first step to achieve the development of such kind of cell culture materials where FGF-2, TGF-β and insulin are conjugated. Furthermore, FGF-2 immobilization on cell culture materials might maintain high activity in the culture medium at 37°C for a long time, which might allow a lower concentration of FGF-2 in the cell culture medium, which is typically used at high temperatures such as 37°C. However, we should consider the design of base materials and reaction for the optimal development of FGF-2-conjugated materials for human PS cell culture and differentiation.

In this study, we designed and prepared two types of materials conjugated to FGF-2 via PEG linker segments using PEG4-SPDP (succinimidyl 3-(2-pyridyldithio)propionate) to effectively cultivate human PS cells. Human PS cells were cultured on FGF-2-immobilized materials in culture medium lacking FGF-2, which was exchanged every day on the same FGF-2-conjugated materials for 6 days per passage. The pluripotency and differentiation ability of human PS cells into cardiomyocytes and retinal pigment epithelium (RPE) were evaluated after long-term (10 passages) culture on FGF-2-conjugated biomaterials.

## Materials and methods

We obtained the approval of the animal experiments from National Central University (NCU-109-010). Each experimental study was done by following all relevant and applicable institutional and governmental guidelines as well as regulations.

### Materials

Human iPS cells (HPS0077) were obtained from Riken BioResource Center (Tsukuba, Japan). The proteins and other materials utilized in this project are tabulated in Supplementary Table S1. The other biomaterials and chemicals utilized in this investigation were supplied by Sigma‒Aldrich (St. Louis, MO, USA).

### Preparing method of PVAI-C hydrogels conjugated with FGF-2

**Figure 1.**
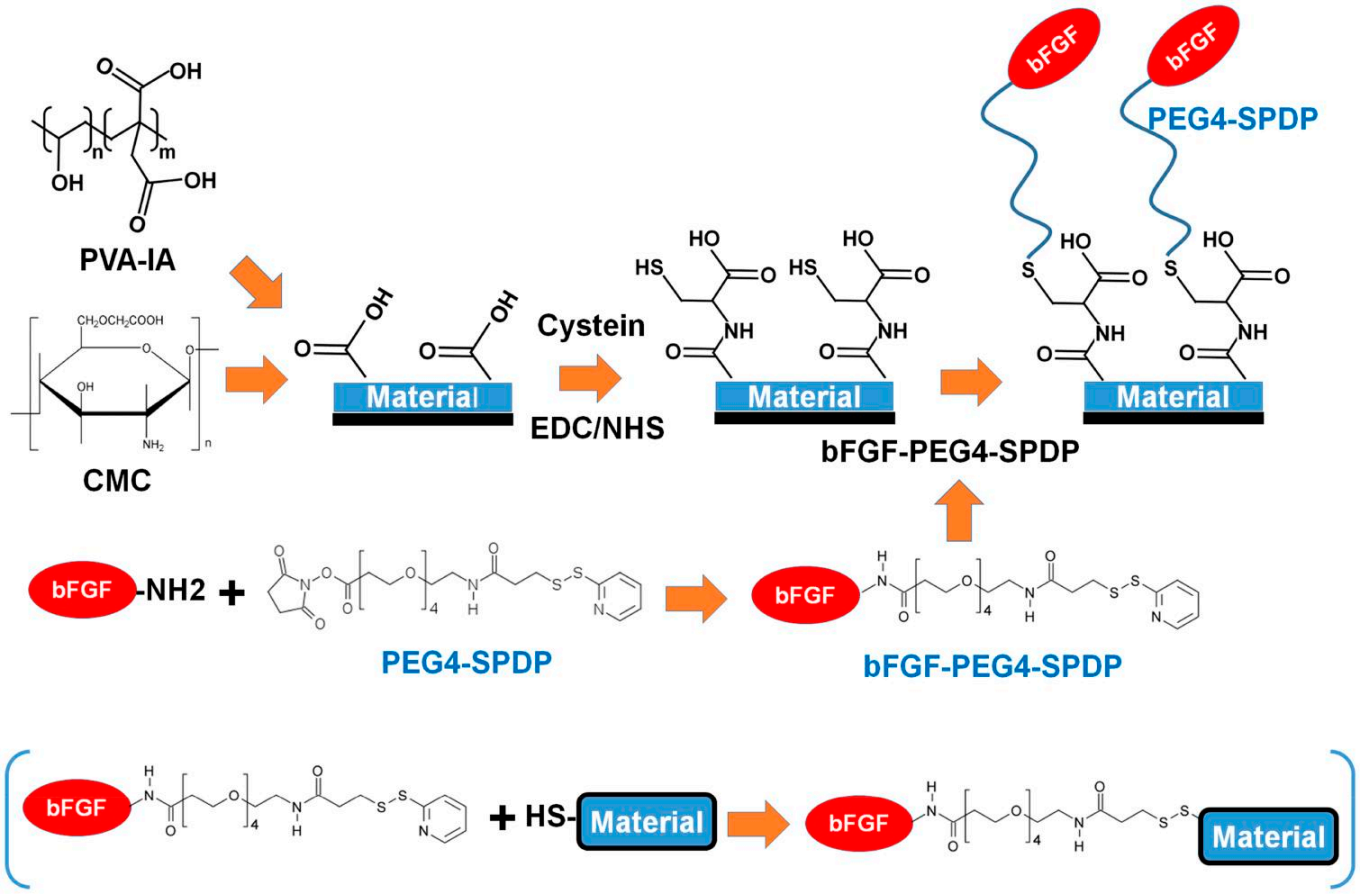
Design of FGF-2-conjugated cell culture biomaterials. PVAI hydrogels and CMC-coated dishes were prepared. Subsequently, the carboxylic acid of the biomaterials was activated using the NHS/EDC reaction, and cysteine was conjugated on the surface of the biomaterials. FGF-2 was reacted with PEG4-SPDP, and FGF-2-conjugated PEG4-SPDP was reacted with cysteine-conjugated biomaterials using the Michael addition reaction. Finally, FGF-2-conjugated biomaterials, PVAI-C-FGF hydrogels and CMC-C-FGF dishes were generated.

The chemical scheme for the formation of poly(vinyl alcohol-co-itaconic acid), PVAI, hydrogel-conjugated FGF-2 is outlined in Figure 1. The base hydrogels were prepared from PVAI with a 98% degree of hydrolysis and 1.3 mol% itaconic acid, in accordance with the protocol used in our previous studies [40–42]. 0.05 wt% PVAI solution was injected into tissue culture polystyrene (TCPS) dishes and dried to form transparent PVAI sheets. The PVAI hydrogels were generated by crosslinking the PVAI sheets in a glutaraldehyde solution (crosslinking solution) (1 w/v%) containing H_2_SO_4_ (1 w/v%) and Na_2_SO_4_ (20 w/v%). The crosslinking degree of the PVAI hydrogels could be controlled by the crosslinking time, which was chosen as 24 h based on the optimal stiffness for human PS cell culture and proliferation determined in our previous works in this project [40–42].

Carboxylic acid functional groups on PVAI hydrogels were activated via immersion in NHS (N-hydroxysuccinimide, 10 mg/ml)/EDC (1-ethyl-3-(3-dimethylaminopropyl)carbodiimide, 10 mg/ml) solution for 60 min at 37°C. After activation, 500 μg/ml cysteine solution was introduced into PVAI hydrogels and incubated overnight at 4°C with gentle shaking (Figure 1). After conjugation of cysteine on PVAI (PVAI-C) hydrogels, PVAI-C hydrogels were rinsed with PBS (phosphate buffered saline) and preserved in PBS at 4°C until the next step.

PEG4-SPDP (20 mM) in PBS and 0.5, 2 or 8 μg/ml FGF-2 in PBS were mixed and incubated at 4°C for half an hour with gentle shaking. After the reaction (Figure 1), the FGF-2 and PEG4-SPDP conjugated complex solution were introduced into the PVAI-C hydrogels and incubated at 4°C overnight with gentle shaking to form PVAI-C hydrogels conjugated with FGF-2. PVAI-C-5FGF, PVAI-C-20FGF and PVAI-C-80FGF indicate FGF-2-conjugated PVAI-C hydrogels prepared with 0.5, 2 and 8 μg/ml FGF-2 solution, respectively. Subsequently, the PVAI-C-5FGF, PVAI-C-20FGF and PVAI-C-80FGF hydrogels were immersed in 5 μg/ml rVN and incubated at 4°C overnight to obtain rVN-coated PVAI-C-FGF (PVAI-C-5FGF, PVAI-C-20FGF and PVAI-C-80FGF) hydrogels.

### Preparation of CMC-C dishes conjugated with FGF-2

For this purpose, 500 μg/ml O-carboxymethyl chitosan (CMC) was introduced into TCPS dishes and incubated at 5°C overnight. Subsequently, TCPS dishes were rinsed with PBS four times to prepare CMC-coated dishes (Figure 1).

Carboxylic acid functional groups on CMC-coated dishes were activated via immersion in EDC/NHS (10 mg/ml) solution for 60 min at 37°C. After activation, 500 μg/ml cysteine solution was inserted into CMC-coated dishes and incubated overnight at 4°C with gentle shaking. After the conjugation of cysteine on CMC-coated (CMC-C) dishes, the CMC-C dishes were rinsed with PBS and stocked in PBS at 4°C until the next step (Figure 1).

PEG4-SPDP (20 mM) in PBS and 0.5, 2 or 8 μg/ml FGF-2 in PBS were mixed and located at 4°C for half an hour with gentle shaking. After the reaction (Figure 1), the FGF-2 and PEG4-SPDP conjugated complex solution was introduced into the CMC-C dishes and incubated at 4°C overnight with gentle shaking to form CMC-C dishes conjugated with FGF-2. CMC-C-5FGF, CMC-C-20FGF and CMC-C-80FGF indicate FGF-2-conjugated CMC-C dishes prepared with 0.5, 2 and 8 μg/ml FGF-2, respectively. CMC-C-FGF dishes were stored at 4°C until cell culture was performed. One day before CMC-C-FGF dishes were used for human iPS cell cultivation, CMC-C-5FGF, CMC-C-20FGF and CMC-C-80FGF dishes were immersed in 5 μg/ml rVN and incubated at 4°C overnight to obtain rVN-coated CMC-C-FGF (CMC-C-5FGF, CMC-C-20FGF and CMC-C-80FGF) dishes.

### Surface assay of PVAI-C hydrogels and CMC-C dishes conjugated with FGF-2

The surface analysis of PVAI-C hydrogels and CMC-C dishes conjugated with FGF-2 was analysed using XPS, X-ray photoelectron spectroscopy (Sigma Probe, Thermo VG-Scientific, UK) to determine the surface chemical composition of the PVAI-C hydrogels or CMC-C dishes conjugated with FGF-2, where a BE (binding energy) of 284.6 eV was selected as a standard peak maximum of the C1s spectra [43–45].

### Human iPS cell culture on PVAI-C hydrogels and CMC-C dishes conjugated with FGF-2

Human iPS cells (HPS0077) were expanded on rVN-coated TCPS surfaces in xeno-free Essential 8 (E8) media [40, 46–49]. Human iPS cells were seeded on rVN-coated PVAI-C hydrogels or CMC-C dishes conjugated with FGF-2 in Essential 8 (E8) medium or E8-minus FGF-2 (Essential 6 medium plus TGF-β1, hereafter denoted Essential 7 (E7) media), at a seeding density of 5 × 10^4^ cells per cm^2^. After human iPS cell culture for 6 days until approximately 84–86% confluence, human iPS cells were passaged on new PVAI-C hydrogels or CMC-C dishes conjugated with FGF-2 utilizing a conventional protocol. The media were changed daily with fresh media for each experiment.

### Human iPS cell pluripotency and expansion

During human iPS cell proliferation on PVAI-C hydrogels or CMC-C dishes conjugated with FGF-2 in medium with or without FGF-2, the expansion fold was calculated using the following equation [46, 47, 49–52]:


(1) Expansion fold ratio=(the number of human iPS cells in the dishes after proliferation)/(the number of human iPS cells in the dishes before proliferation).

The cells of differentiation were assayed from SSEA-4 expression using flow cytometry (BD Accuri™ C6, BD Biosciences, Franklin Lakes, NJ, USA). The following calculation was used to estimate the differentiated rate of human iPS cells after proliferation on PVAI-C hydrogels or CMC-C dishes conjugated with FGF-2 at each passage:


(2) The differentiated rate (%)=100%−SSEA-4 expression of human iPScells  (%)

The pluripotent protein expression of human iPS cells was investigated by immunohistochemical staining using specific antibodies against the pluripotency proteins Oct3/4, Nanog, Sox-2 and SSEA-4, following a conventional method [40, 41, 47, 49–53]. The antibodies used in this study are also summarized in Supplementary Table S1. The stained cells were observed under a fluorescence microscope (Eclipse Ti-U fluorescence inverted microscope, Nikon Instruments, Inc., Tokyo, Japan) or confocal laser microscopy (LSM900 with Airyscan, Zeiss, Aalen, Germany).

Karyotype was evaluated using a conventional method [54, 55].

### EB formation

The embryoid body (EB) generation of human iPS cells was proceeded using a conventional protocol [40, 41, 47, 49–52, 56].

After EB generation from human iPS cells proliferated on rVN-coated PVAI-C hydrogels or CMC-C dishes conjugated with FGF-2 in medium without FGF-2 for 10 passages, the cells were evaluated by immunohistochemical staining using antibodies against markers of three embryonic germline layers (endoderm cells: AFP (α-fetoprotein), mesoderm cells: SMA (smooth muscle actin) and ectoderm cells: GFAP (glial fibrillary acidic protein)) using a conventional protocol, and the differentiated cells were investigated [40, 41, 47, 49–52]. The antibodies used in this study are also summarized in Supplementary Table S1.

### Teratoma formation

Teratoma formation assays, which can verify the potential of human iPS cells to differentiate into cells derived from all three germ layers, were performed following a conventional protocol [40, 41, 47, 49–52, 56, 57] after human iPS cells were proliferated on PVAI-C hydrogels or CMC-C dishes conjugated with FGF-2 in medium without containing FGF-2 for 10 passages. In brief, 7 weeks after subcutaneous transplantation of human iPS cells into NOD.CB17-Prkdcscid/JNarl, NOD/SCID (nonobese diabetic/severe combined immunodeficiency), mice and teratomas were typically created. Then, the teratomas were cut and fixed in a formaldehyde solution. The paraffin-embedded teratomas were carefully prepared and sectioned in the hospital and stained with H&E (hematoxylin and eosin) solution by conventional procedures [40, 41, 47, 49–52].

### Human iPS cell differentiation into cardiomyocytes

Human iPS cells were induced to differentiate into cardiac cells utilizing a protocol developed by Sharma *et al.* [58] with some modifications after culture on rVN-coated PVAI hydrogels or CMC-C dishes conjugated with FGF-2 in medium without FGF-2 for 10 passages.

On day -5, human iPS cells were inoculated into rVN-coated PVAI hydrogels or CMC-C dishes conjugated with FGF-2 and cultured in E6 medium supplemented with TGF-β1 until day 0, and the media were exchanged every day. On day 0, human iPS cells typically appeared to be around 75–85% confluent. The medium was replaced with RPMI 1640 medium supplemented with B27 minus insulin (2 wt%) and a GSK-3β inhibitor (6 µM CHIR99021). Human iPS cells were cultured for another two days. On day 2, the media were exchanged with RPMI 1640 medium supplemented with 2 wt% B27 minus insulin, and the cells were incubated for another two days. On day 4, the media were replaced with RPMI 1640 media supplemented with IWR-1 (5 µM, a Wnt inhibitor) and B27 minus insulin (2 wt%), and the cells were incubated for an additional two days. On day 6, the media were replaced with RPMI 1640 media supplemented with B27 minus insulin (2 wt%), and the cells were incubated for another day. On day 7, the media were replaced with RPMI 1640 medium supplemented with B27 (2 wt%). The cells (cardiomyocytes derived from human iPS cells) were cultivated for three weeks, and the media were replaced with new cultivation media every two days.

### Characterization of human iPS cell-derived cardiac cells

The expression of troponin T (cTnT, cardiac marker) on cardiomyocytes derived from human iPS cells was assayed utilizing flow cytometry followed by a conventional protocol with a primary antibody solution of an isotype antibody (IgG1 isotype-mouse antibody) solution (1:200 dilution) or cTnT (1:200 dilution) antibody solution and an secondary antibody solution (Alexa Fluor 488 goat anti-mouse IgG, 1:1000 dilution) [59]. The antibodies used in this study are also summarized in Supplementary Table S1.

Immunohistochemical staining of cardiac cells differentiated from human iPS cells was proceeded for cardiomyocyte marker proteins (cTnT, ML2Cv, α-actinin and NKX2.5) utilizing conventional techniques, where primary antibodies against cTnT (1:200 dilution, green), ML2Cv (1:200 dilution, red) α-actinin (1:200 dilution, red or green) and NKX2.5 (1:200 dilution, red) and secondary antibodies of Alexa Fluor 488 goat anti-mouse IgG antibody (1:500 dilution), Alexa Fluor 555 goat anti-rabbit IgG antibody (1:500 dilution) and Alexa Fluor 594 donkey anti-mouse IgG (1:500 dilution) were used [59]. The expression of ML2Cv, cTnT, α-actinin and NKX2.5 as well as the nuclei with staining by DAPI were evaluated with confocal laser microscopy [59].

### Human iPS cell differentiation into RPE

Human iPS cells were also induced to differentiate into RPE utilizing a protocol reported by Smith *et al*. [60] and Maruotti *et al*. [61] with several modifications after culture on CMC-C dishes conjugated with FGF-2 in medium without FGF-2 for 10 passages. In brief, human iPS cells were proliferated in mTESR1 media until the cells became confluence. Subsequently, the cultivation media were changed to RPE differentiation media (RDM), and this time spot was the first day (D1). The RDM (100 ml) was made of 15 ml KSR (KnockOut Serum Replacement) and 85 ml DMEM/F12 with supplementation of 1 ml MEM NEAA (nonessential amino acids, 100×), 1 ml glutamine (100×), 1 ml anti–anti (antibiotic–antimycotic, 100×) and 0.7 μl β-mercaptoethanol. At D2 to D14, 10 mM NIC (nicotinamide) and 10–50 nM CTM (chetomin), which was enhanced 10 nM from 10 nM to 50 nM every 3 days, were also included to the RDM. After D14, RDM supplemented with only 10 mM NIC was utilized, and the media were exchanged with fresh RDM every day for another two weeks. On D28, the media were replaced by RPE media until mature and pigmented RPE cells were detected. The RPE media (100 ml) was made of 30 ml F12 and 70 ml DMEM, which was supplemented with 1 ml anti–anti (100×) and 2 ml B27 (50×).

### Characterization of human iPS cell-derived RPE cells

The expression of RPE65 (RPE marker) on RPE cells differentiated from human iPS cells was evaluated utilizing flow cytometry followed by a conventional protocol with a primary antibody solution of RPE65 (1:200 dilution) or an isotype antibody (IgG1 isotype-mouse antibody) solution (1:200 dilution) and a secondary antibody (Alexa Fluor 488 goat anti-mouse IgG) solution (1:1000 dilution).

Immunohistochemical staining of RPE cells differentiated from human iPS cells was performed for RPE marker proteins (PAX6, MITF, ZO-1 and RPE65) utilizing conventional techniques, where primary antibodies against PAX6 (1:200 dilution, green), MITF (1:200 dilution, red), ZO-1 (1:200 dilution, red) and RPE65 (1:200 dilution, green) and secondary antibodies of Alexa Fluor 488 goat anti-mouse IgG antibody (1:500 dilution) and Alexa Fluor 555 goat anti-rabbit IgG antibody (1:500 dilution) were used [59]. The expression of PAX6, MITF, ZO-1 and RPE65 as well as the nuclei stained with DAPI were assayed with a confocal laser microscopy [59].

### Statistical analysis

The data from four samples were analysed in this project. The data are shown as the means ± standard deviations. The statistical evaluation was performed utilizing one-way ANOVA with a *post hoc t* test. The Tukey‒Kramer *post hoc* test was also proceeded after one-way ANOVA. Probability (*P*) values less than 0.05 were regarded statistically significant.

## Results and discussion

### Preparation of FGF-2-conjugated cell culture biomaterials

We designed two different types of FGF-2-conjugated biomaterials: PVAI-C hydrogels conjugated with FGF-2 (PVAI-C-5FGF, PVAI-C-20FGF and PVAI-C-80FGF) and CMC-C dishes conjugated with FGF-2 (CMC-C-5FGF, CMC-C-20FGF and CMC-C-80FGF). PVAI hydrogels grafted with several designed peptides were prepared and found to be excellent cell culture materials for human PS cell culture and differentiation [40, 41, 62, 63], although previous works used E8 medium, which contains unstable FGF-2. Therefore, we prepared PVAI hydrogels conjugated with FGF-2 in which human iPS cells can be cultured in E7 medium, which does not contain unstable FGF-2. The chemical scheme of the formation of PVAI hydrogels conjugated with FGF-2 is shown in Figure 1. The carboxyl group of PVAI hydrogels was activated with EDC/NHS solution. Subsequently, cysteine was conjugated on PVAI hydrogels to create a thiol group on PVAI (PVAI-C) hydrogels, whereas the amino group of FGF-2 was conjugated with the crosslinking agent PEG4-SPDP [64]. PEG4-SPDP-activated FGF-2 was reacted with the thiol group of PVAI hydrogels using Michael addition (Click reaction) to generate PVAI hydrogels conjugated with FGF-2 (PVAI-C-5FGF, PVAI-C-20FGF and PVAI-C-80FGF), which are denoted as PVAI-C-FGF hydrogels.

FGF-2 was also conjugated to CMC biomaterials (CMC-C-5FGF, CMC-C-20FGF and CMC-C-80FGF). This is because the carboxylic acid content on CMC is much higher than that on PVAI, which may lead to a higher FGF-2 conjugated density on CMC-based biomaterials than on PVAI-based hydrogels. The chemical scheme of the preparation of CMC-coated dishes conjugated with FGF-2 is also shown in Figure 1. First, CMC-coated dishes were prepared from CMC coating on the dishes. Subsequently, CMC-coated dishes were activated with EDC/NHS solution. Then, the cysteine was conjugated to CMC-coated dishes to create a thiol group on CMC-coated (CMC-C) dishes. After FGF-2 was activated with crosslinker of PEG4-SPDP, PEG4-SPDP-activated FGF-2 was reacted with the thiol group of CMC-C dishes using Michael addition (Click reaction) to generate CMC-C dishes conjugated with FGF-2 (CMC-C-5FGF, CMC-C-20FGF and CMC-C-80FGF) (Figure 1).

### Physical characteristics of PVAI-C hydrogels conjugated with FGF-2

The surfaces of PVAI-C hydrogels conjugated with FGF-2 (PVAI-C-FGF hydrogels) and without FGF-2 (PVAI and PVAI-C hydrogels) were evaluated using XPS. This characterization confirmed the presence of FGF-2 on the PVAI-C-FGF hydrogel surfaces. This is because the PVAI hydrogels were composed solely of hydrogen, oxygen and carbon atoms with no nitrogen and/or sulfur atoms, attributing to the amino acids of FGF-2 and/or cysteine. The high-resolution XPS spectra of the S2p, N1s and C1s peaks on the surfaces of the PVAI, PVAI-C, PVAI-C-5FGF, PVAI-C-20FGF and PVAI-C-80FGF hydrogels are depicted in Supplementary Figure S1. The PVAI-C-5FGF, PVAI-C-20FGF and PVAI-C-80FGF hydrogels showed broader C1s peaks than the PVAI hydrogels, which were not conjugated with FGF-2. The N1s peaks of the PVAI hydrogels conjugated with FGF-2 (PVAI-C-FGF hydrogels) were more significant than those of the PVAI and PVAI-C hydrogels, which were not conjugated with FGF-2. The existence of FGF-2 on PVAI-C-FGF hydrogels contributed to the enhanced N1s peaks compared to those of PVAI and PVAI-C hydrogels. In particular, PVAI hydrogels consist solely of carbon, oxygen and hydrogen, with no sulfur or nitrogen atoms, and the enhanced N1s peak on PVAI-C-FGF hydrogels can be safely attributed to the presence of FGF-2 on the PVAI-C-FGF hydrogels (PVAI-C-5FGF, PVAI-C-20FGF and PVAI-C-80FGF). These observations indicate that FGF-2 was conjugated onto the PVAI-C-FGF hydrogels.

**Figure 2.**
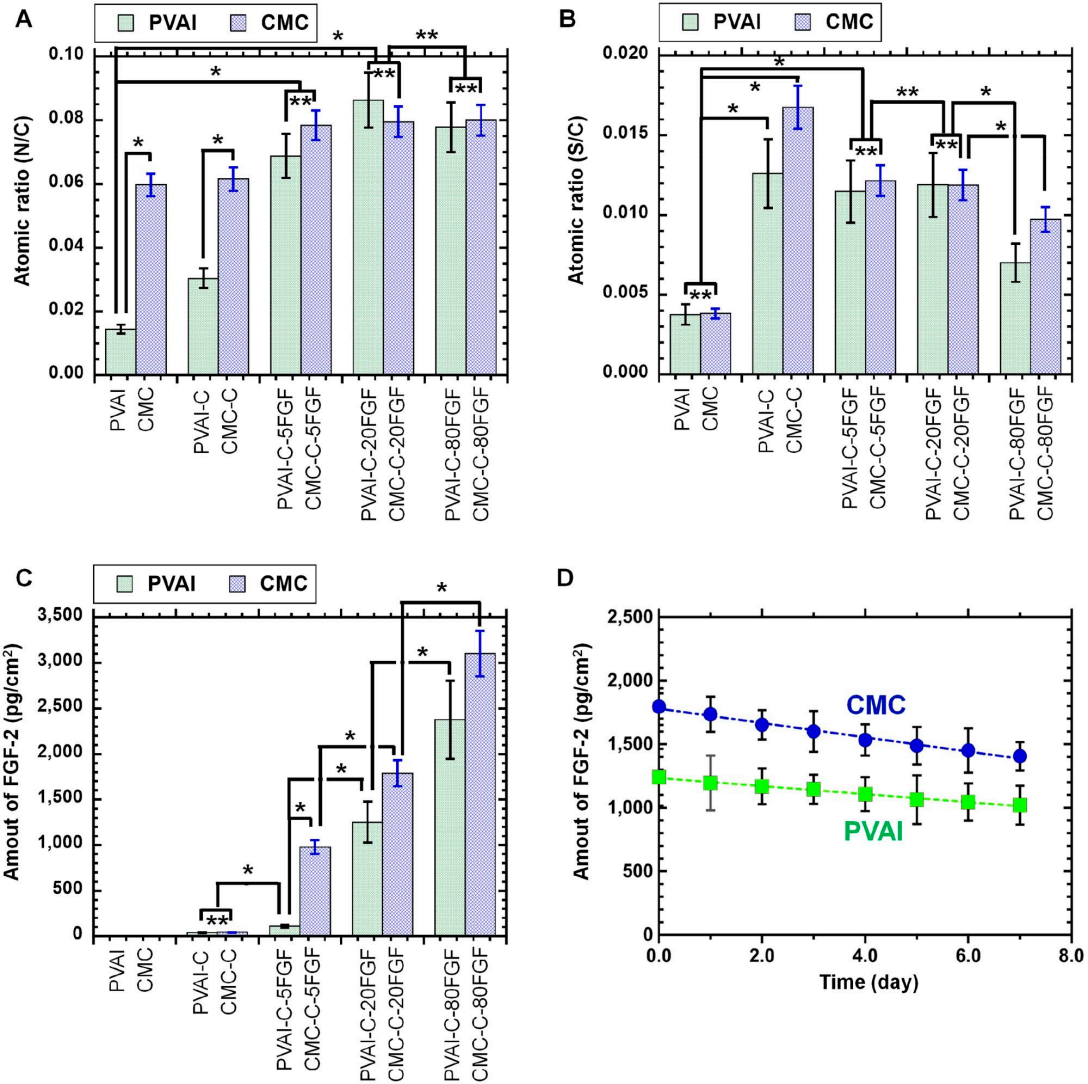
Physical characteristics of the PVAI hydrogel surface and CMC-coated dishes with and without FGF-2 conjugation. (**A**) Nitrogen to carbon (N/C) atomic ratios of the PVAI hydrogel surface (left bar) and CMC-coated dishes (right bar) with and without conjugation of FGF-2, which were analysed by XPS. * *P *<* *0.05. ** *P *>* *0.05. (**B**) Sulfur to carbon (S/C) atomic ratios of the surfaces of PVAI hydrogels (left bar) and CMC-coated dishes (right bar) with and without conjugation of FGF-2, which were analysed by XPS. * *P *<* *0.05. ** *P *>* *0.05. (**C**) Surface density of FGF-2 conjugated on the surfaces of PVAI hydrogels (left bar) and CMC-coated dishes (right bar) with and without FGF-2 conjugation, which were analysed by ELISA. * *P *<* *0.05. ** *P *>* *0.05. (**D**) Time dependence of FGF-2 density conjugated on the surfaces of PVAI hydrogels (closed square) and CMC-coated dishes (closed circle) with FGF-2 conjugation, which were immersed in PBS buffer solution for 7 days.

The N/C atomic ration and S/C atomic ratio of the surfaces of PVAI, PVAI-C and PVAI-C-FGF were calculated and are shown in Figure 2A and B, respectively. The N/C ratio of the PVAI-C-FGF hydrogel surface ranged from 0.7 to 0.9, which was extensively higher than the N/C ratio of the PVAI hydrogels without FGF-2 (*P *<* *0.05). The S/C ratio on the PVAI-C-FGF hydrogel surface was higher than that of the PVAI hydrogels but was similar to that of the PVAI-C hydrogels. This is explained by the fact that PVAI-C hydrogels contain cysteines, which include sulfur groups in the amino acid of cysteine. There was no extensive difference in the S/C ratio between PVAI-C-5FGF and PVAI-C-20FGF (*P *>* *0.05), and the S/C ratio on PVAI-C-80FGF seemed to be lower than that on PVAI-C-5FGF and PVAI-C-20FGF. Currently, the exact explanation for why PVAI-C-80FGF showed a lower S/C ratio but a similar N/C ratio compared to PVAI-C-5FGF and PVAI-C-20FGF is unknown, but one possible explanation is that FGF-2 might aggregate at higher concentrations, such as 80 µg/ml, but not at 5 or 20 µg/ml, and aggregated FGF-2 does not readily conjugate to the PVAI-C hydrogel surface.

ELISA was performed to quantitate FGF-2 on PVAI-C-FGF hydrogels after the PVAI-C-FGF hydrogels were prepared, and the results are shown in Figure 2C. The surface density of FGF-2 enhanced with the concentration of FGF-2 solution, which was conjugated to PVAI-C-FGF hydrogels using PEG4-SPDP as a crosslinker, whereas the surface density of FGF-2 on PVAI and PVAI-C hydrogels was measured at trace concentrations (<40 pg/cm^2^). In particular, the surface density of FGF-2 on PVAI-C-20FGF and PVAI-C-80FGF hydrogels was observed to be approximately 1250 and 2400 pg/cm^2^, respectively, from ELISA.

One passage of human iPS cell culture is typically 5 days. Therefore, time dependence of the amount of FGF-2 on PVAI-C-20FGF hydrogels in PBS buffer solution (pH 7.4) was evaluated for 7 days and the results are depicted in Figure 2D. FGF-2 was immobilized on PVAI hydrogels with only 15% releasing from the FGF-2 conjugated surface for 5 days.

The FGF-2 concentration in Essential 8 medium is reported to be 0.1 µg/ml, which corresponds to 15 625 pg/cm^2^ for 1.5 ml of E8 medium in a 6-well cell proliferation plate with a surface area of 9.6 cm^2^. The FGF-2 immobilization amount on the PVAI-C-20FGF and PVAI-C-80FGF hydrogels was 12.5/day × 6 days (six culture medium changes for one passage) = 75 times and 6.5 times/day × 6 days = 39 times less than the usage of FGF-2 in culture medium for one passage (6 days) of human iPS cell proliferation, respectively.

### Characterization of CMC-C dishes conjugated with FGF-2

The surfaces of CMC-C dishes conjugated with FGF-2 (CMC-C-FGF dishes) and CMC and CMC-C dishes without FGF-2 were also evaluated utilizing XPS. This characterization was also useful to identify the presence of FGF-2 on the CMC-C-FGF dish surfaces and to compare the amount of FGF-2 on CMC-C-FGF dishes and PVAI-C- FGF hydrogels. The high-resolution XPS spectra of the S2p, N1s and C1s peaks on the surfaces of the CMC, CMC-C, CMC-C-5FGF, CMC-C-20FGF and CMC-C-80FGF dishes are shown in Supplementary Figure S2.

The N/C and S/C atomic ratios of the CMC, CMC-C and CMC-C-FGF surfaces were calculated and are also shown in Figure 2A and B, respectively. The N/C ratio on the CMC-C-FGF surface showed similar N/C ratios to the surfaces on CMC and CMC-C dishes. The S/C ratio of the CMC-C-FGF surfaces was higher than the surfaces of the CMC and CMC-C hydrogels. There was no extensive difference in the S/C ratio between CMC-C-5FGF and CMC-C-20FGF (*P *>* *0.05), and the S/C ratio on CMC-C-80FGF seemed to be lower than that on CMC-C-5FGF and CMC-C-20FGF. This is the same tendency of the S/C ratio among the PVAI-FGF hydrogels. The exact explanation for why CMC-C-80FGF showed a lower S/C ratio than CMC-C-5FGF and CMC-C-20FGF is unknown, but one possible explanation is that FGF-2 might aggregate at higher concentrations, such as 80 µg/ml, but not 5 or 20 µg/ml, and aggregated FGF-2 does not readily conjugate to the CMC-C hydrogel surface.

ELISA was performed to investigate the quantitative amount of FGF-2 on CMC, CMC-C and CMC-C-FGF dishes after CMC-C-FGF dishes were prepared, and the results are shown in Figure 2C. The surface density of FGF-2 enhanced with the concentration of FGF-2 solution used for conjugation to CMC-C-FGF dishes using PEG4-SPDP as a crosslinker, whereas the surface density of FGF-2 on CMC and CMC-C dishes was measured at trace concentrations (<50 pg/cm^2^). In particular, the surface density of FGF-2 on CMC-C-5FGF, CMC-C-20FGF and CMC-C-80FGF dishes was observed to be approximately 1000, 1800 and 3100 pg/cm^2^, respectively, by ELISA. The FGF-2 concentration in Essential 8 medium is reported to be 0.1 µg/ml, which corresponds to 15 625 pg/cm^2^ for 1.5 ml of E8 medium used in a 6-well cell proliferation plate with a surface area of 9.6 cm^2^. The amount of FGF-2 immobilized on CMC-C-5FGF, CMC-C-20FGF and CMC-C-80FGF dishes was 15.6/day × 6 days (six culture medium changes for one passage) = 93.6 times, 8.7/day × 6 days (six culture medium changes for one passage) = 52.2 times and 5.0/day × 6 days (six culture medium changes for one passage) = 30 times less than the usage of FGF-2 in culture medium for one passage (6 days) of human iPS cell culture, respectively.

One passage of human iPS cell culture is typically 5–7 days. Therefore, time dependence of the amount of FGF-2 on CMC-C-20FGF dishes in PBS buffer solution (pH 7.4) was evaluated for 7 days and the results are depicted in Figure 2D. FGF-2 was immobilized on CMC-C-20FGF dishes with only 17% releasing from the FGF-2 conjugated surface for 5 days.

The amount of FGF-2 on CMC-C-FGF dishes was higher than that on PVAI-C-FGF when these cell culture biomaterials were prepared with the same concentration of FGF-2 (*P *<* *0.05).

### Human iPS cell culture on rVN-coated PVAI-C hydrogels conjugated with FGF-2

**Figure 3.**
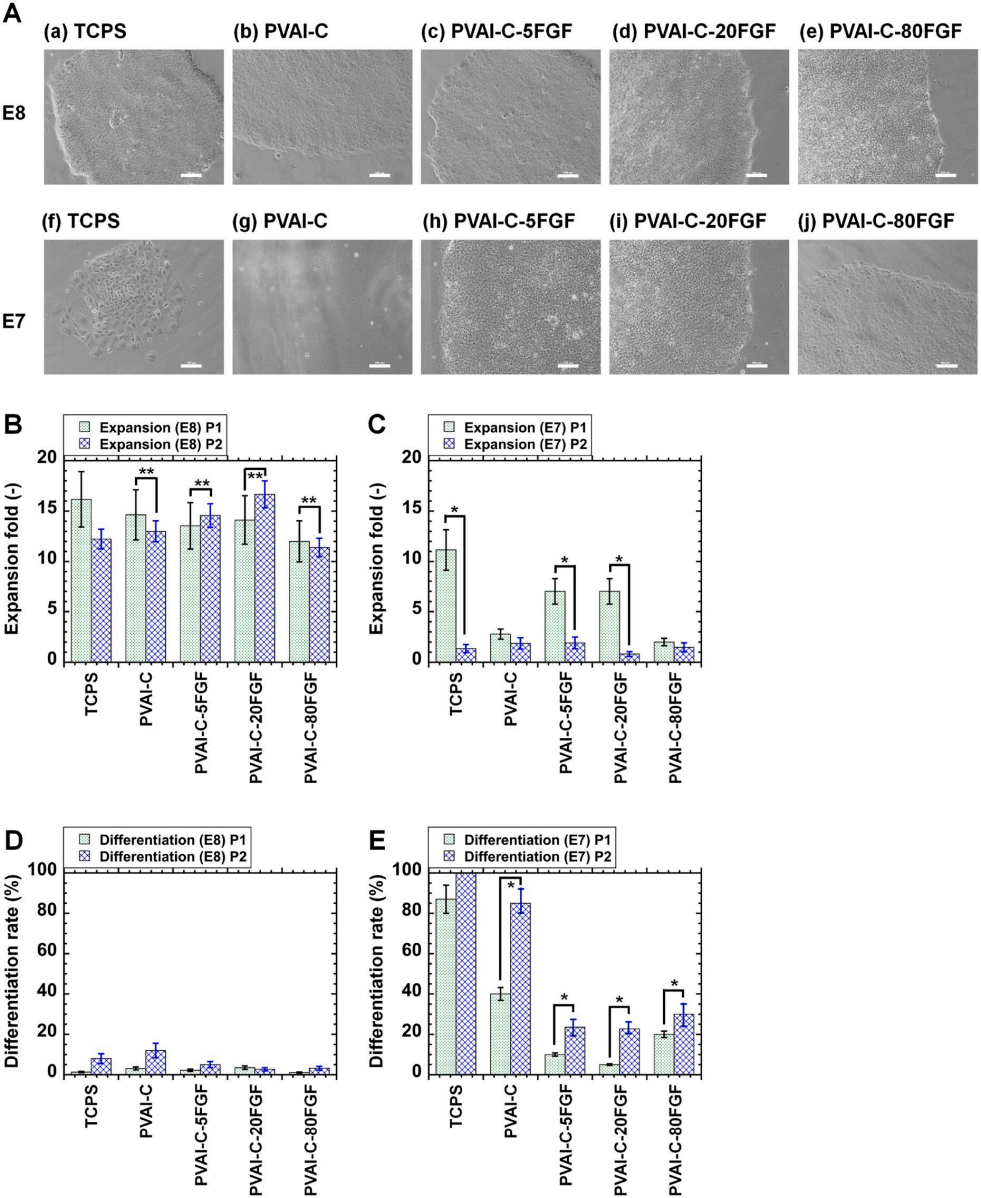
Human iPS cell (HPS0077) culture on PVAI hydrogels conjugated with and without FGF-2 in the E8 and E7 medium, which contains and not contains FGF-2, respectively under xeno-free culture methods. (**A**) Morphologies of human iPS cells on rVN-coated TCPS (TCPS) dishes (**a**, **f**), rVN-coated PVAI-C hydrogels (**b**, **g**), rVN-coated PVAI-C-5FGF hydrogels (**c**, **h**), rVN-coated PVAI-C-20FGF hydrogels (**d**, **i**) and rVN-coated PVAI-C-80FGF hydrogels (**e**, **j**) in E8 medium (**a**–**e**) and E7 medium (**f**–**j**) at passage 1. The scale bar describes 100 μm. (**B**) Expansion fold of human iPS cells on rVN-coated TCPS (TCPS) dishes, rVN-coated PVAI-C hydrogels, rVN-coated PVAI-C-5FGF hydrogels, rVN-coated PVAI-C-20FGF hydrogels and rVN-coated PVAI-C-80FGF hydrogels at passage 1 (left bar) and passage 2 (right bar) in E8 medium, which contains FGF-2. (**C**) Expansion fold of human iPS cells on rVN-coated TCPS (TCPS) dishes, rVN-coated PVAI-C hydrogels, rVN-coated PVAI-C-5FGF hydrogels, rVN-coated PVAI-C-20FGF hydrogels and rVN-coated PVAI-C-80FGF hydrogels at passage 1 (left bar) and passage 2 (right bar) in E7 medium, which does not contain FGF-2. (**D**) Differentiation rate of human iPS cells on rVN-coated TCPS (TCPS) dishes, rVN-coated PVAI-C hydrogels, rVN-coated PVAI-C-5FGF hydrogels, rVN-coated PVAI-C-20FGF hydrogels and rVN-coated PVAI-C-80FGF hydrogels at passage 1 (left bar) and passage 2 (right bar) in E8 medium, which contains FGF-2. (**E**) Differentiation rate of human iPS cells on rVN-coated TCPS (TCPS) dishes, rVN-coated PVAI-C hydrogels, rVN-coated PVAI-C-5FGF hydrogels, rVN-coated PVAI-C-20FGF hydrogels and rVN-coated PVAI-C-80FGF hydrogels at passage 1 (left bar) and passage 2 (right bar) in E7 medium, which does not contain FGF-2.

Human iPS cells (HPS0077) were cultivated for two passages on rVN-coated TCPS dishes, rVN-coated PVAI-C hydrogels and rVN-coated PVAI-C-FGF (PVAI-C-5FGF, PVAI-C-20FGF and PVAI-C80FGF) hydrogels in E8 medium containing FGF-2 and E7 medium not containing FGF-2. The cell morphologies of human iPS cells at passage 1 are depicted in Figure 3A. Human iPS cells were successively cultivated in E8 medium on all cell proliferation biomaterials used in this project (rVN-coated TCPS dishes, rVN-coated PVAI-C hydrogels and rVN-coated PVAI-C-FGF hydrogels). However, only human iPS cells showed healthy cell colonies on PVAI-C-FGF hydrogels in E7 medium, whereas human iPS cells could not attach to the surface or showed single or small colonies with differentiated cell morphologies on rVN-coated TCPS and rVN-coated PVAI-C hydrogels in E7 medium. These results indicate that FGF-2 is essential for human iPS cell proliferation whether or not FGF-2 is in suspension or immobilized on the cell culture material surface.

The expansion fold of human iPS cells proliferated on PVAI hydrogels with and without conjugated FGF-2, which were coated with rVN, as well as on rVN-coated TCPS dishes, was evaluated for two passages in medium containing FGF-2 (E8 medium) and medium without FGF-2 (E7 medium), and the results are depicted in Figure 3B and C, respectively. Human iPS cells can be successfully cultured on rVN-coated PVAI hydrogels conjugated with FGF-2 (PVAI-C-FGF hydrogels) and without FGF-2 (PVA-C hydrogels) as well as rVN-coated TCPS dishes in E8 medium. Furthermore, human iPS cells on rVN-coated TCPS dishes, rVN-coated PVAI-C-5FGF and rVN-coated PVAI-C-20FGF showed more than 7-fold higher expansion in passage 1 in E7 medium. However, human iPS cells on any cell culture material, including PVAI hydrogels conjugated with FGF-2, could not proliferate at passage 2 in E7 medium. This result indicates that FGF-2 on PVAI hydrogels is not active or that the release of FGF-2 from PVAI hydrogels is not sufficient for human iPS cell culture in this study.

The differentiation rate of human iPS cells proliferated on rVN-coated PVAI-C hydrogels conjugated with and without FGF-2 as well as rVN-coated TCPS dishes was also evaluated for two passages in E7 and E8 medium, and the results are described in Figure 3D and E, respectively. As expected, the differentiation rate of human iPS cells on any cell proliferation materials evaluated in this research was less than 15% when the cells were cultured in E8 medium (Figure 3D). The differentiation rate of the cells proliferated on rVN-coated PVAI-C-FGF hydrogels (PVAI-C-5FGF, PVAI-C-20FGF and PVAI-C-80 FGF) was statistically less than that on rVN-coated TCPS dishes and rVN-coated PVAI-C hydrogels in E8 medium (*P *<* *0.05). The differentiation rate of human iPS cells cultured on rVN-coated TCPS dishes and rVN-coated PVAI-C hydrogels was extensively high, such as over 80% at passage 2 in E7 medium, whereas the differentiation rate of human iPS cells proliferated on rVN-coated PVAI-C-FGF hydrogel (PVAI-C-5FGF, PVAI- C-20FGF and PVAI-C-80 FGF) was found to be significantly less than that on rVN-coated TCPS dishes and rVN-coated PVAI-C hydrogels (*P *<* *0.05) (Figure 3E). The conjugation of FGF-2 on PVAI hydrogels contributed to maintaining the pluripotency of human iPS cells more effectively than rVN-coated TCPS and rVN-coated PVAI-C hydrogels. However, human iPS cells could not be cultivated on rVN-coated PVAI-C-FGF hydrogels for more than two passages in this study.

### Human iPS cell culture on rVN-coated CMC-C dishes conjugated with FGF-2

We also prepared rVN-coated CMC-C dishes conjugated with FGF-2 (CMC-C-FGF dishes) because the amount of FGF-2 immobilized on rVN-coated CMC-C-FGF dishes was higher than that on rVN-coated PVAI-C-FGF hydrogels when the same concentration of FGF-2 solution was used for their preparation (Figure 2C). Furthermore, the amount of rVN immobilized on rVN-coated CMC-C-FGF dishes and rVN-coated PVAI-C-FGF hydrogels should be different, as rVN on the cell proliferation biomaterial surface contributes to the adhesion of huma PS cells on the surface [65]. Therefore, we investigated human iPS cell (HPS0077) culture in E8 medium and E7 medium on rVN-coated CMC-C-FGF dishes (CMC-C-5FGF and CMC-C-20FGF) as well as on rVN-coated TCPS dishes and rVN-coated CMC-C dishes as control experiments. To reduce the usage of FGF-2, which is one of the purposes of this research, we did not try to culture human iPS cells on CMC-C-80FGF dishes.

**Figure 4.**
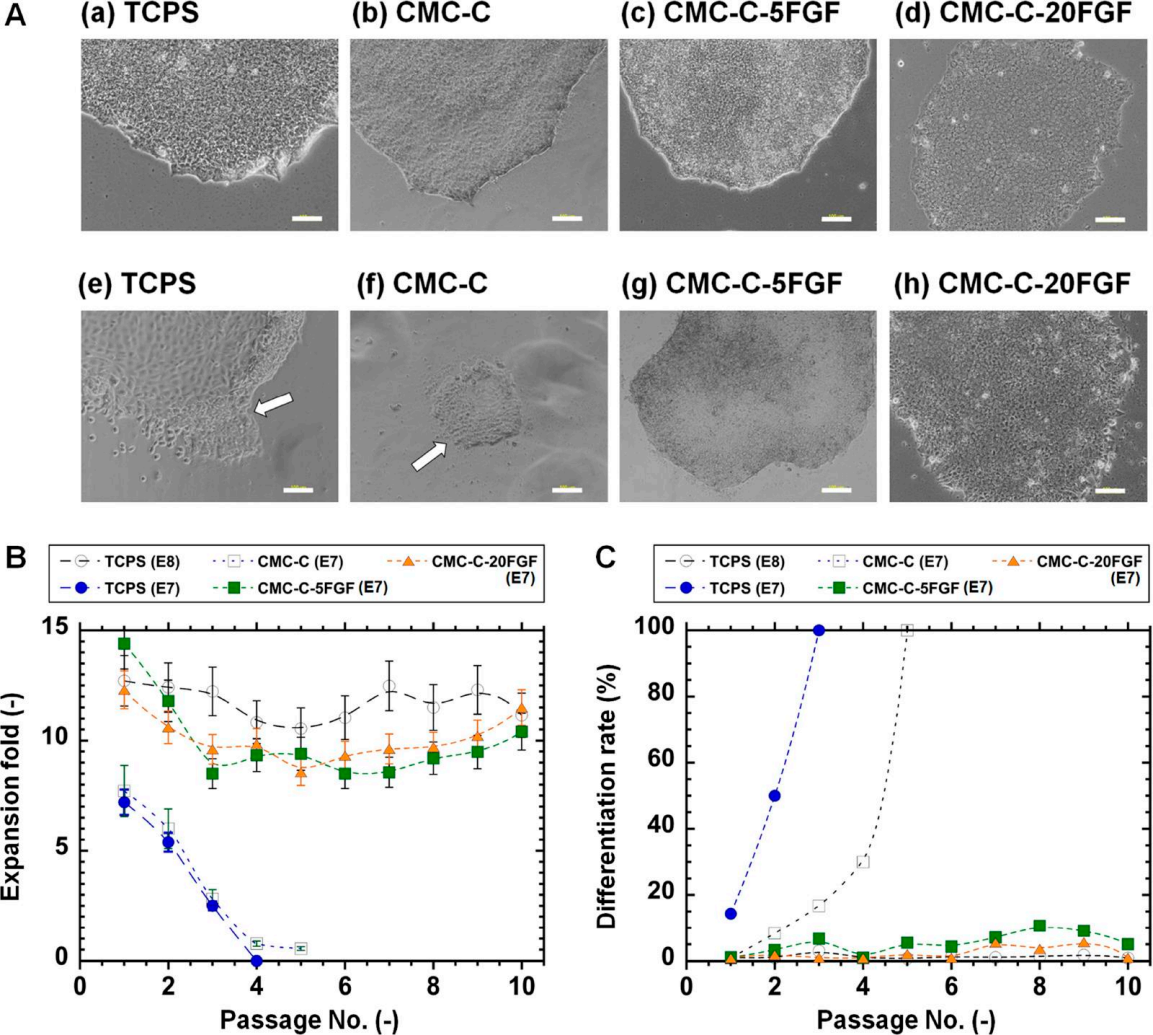
Long-term proliferation of human iPS cells on rVN-coated CMC dishes conjugated with FGF-2 in the E8 medium, which contains FGF-2, and E7 medium, which does not, under xeno-free culture methods. (**A**) Morphologies of human iPS cells on rVN-coated TCPS (TCPS) dishes (**a**, **e**), rVN-coated CMC-C dishes (**b**, **f**), rVN-coated CMC-C-5FGF dishes (**c** and **g**) and rVN-coated CMC-C-20FGF dishes (**d** and **h**) in E8 medium (**a**–**d**) and E7 medium (**e**–**h**) at 3 passages (**e**, **f**) or 10 passages (**a**–**d**, **g**, **h**). The white bar indicates differentiated cells. The scale bar depicts 100 μm. (**B**) Passage dependent of expansion fold of human iPS cells (HPS0077) on rVN-coated TCPS dishes (closed circle), rVN-coated CMC-C dishes (open square), rVN-coated CMC-C-5FGF dishes (closed square) and CMC-C-20FGF dishes (closed triangle) in E7 medium, which does not contain FGF. The data of human iPS cells cultured on rVN-coated TCPS dishes in E8 medium, which contains FGF-2, as a positive control are also shown (open circle). (**C**) Passage dependent of differentiation rate of human iPS cells (HPS0077) on rVN-coated TCPS dishes (closed circle), rVN-coated CMC-C dishes (open square), rVN-coated CMC-C-5FGF dishes (closed square) and CMC-C-20FGF dishes (closed triangle) in E7 medium, which does not contain FGF. The data of human iPS cells cultured on rVN-coated TCPS dishes in E8 medium, which contains FGF-2, as a positive control are also shown (open circle).

The cell morphologies of human iPS cells at passage 3 or 10 are shown in Figure 4A. Human iPS cells were successively cultivated in E8 medium on all cell culture biomaterials, as expected (rVN-coated TCPS dishes, rVN-C-CMC, rVN-CMC-C-5FGF and CMC-C-20FGF). However, human iPS cells could not be cultured on rVN-coated TCPS dishes and rVN-coated CMC-C dishes in E7 medium over passages 3–4, whereas human iPS cells were successfully cultivated on rVN-CMC-C-5FGF and CMC-C-20FGF dishes for 10 passages in E7 medium (Figure 4A).

Figure 4B and C shows the expansion fold and differentiation rate, respectively, of human iPS cells cultured on rVN-coated TCPS dishes as well as rVN-coated C-CMC, CMC-C-5FGF and CMC-C-20FGF dishes for long-term (10 passages) expansion in E7 medium where FGF-2 was depleted from E8 medium. Human iPS cells were successfully cultured on rVN-coated CMC-C-5FGF and rVN-coated CMC-C-20FGF dishes for over 10 passages in E7 media, whereas the expansion fold of human iPS cells on rVN-coated TCPS dishes and rVN-coated CMC-C dishes gradually decreased with increasing passage number in E7 medium (Figure 4B). The differentiation rate of human iPS cells on rVN-coated TCPS dishes and rVN-coated CMC-C dishes gradually increased with increasing passage number (Figure 4C). Human iPS cells were successfully cultured for over 10 passages on rVN-coated CMC-C-5FGF and CMC-C-20FGF dishes in medium without FGF-2 (E7 medium) in this study.

Notably, the amount of FGF-2 immobilized on CMC-C-5FGF dishes was similar to or less than that on PVAI-C- 20FGF and PVAI-C-80FGF hydrogels, respectively. However, human iPS cells could not be cultured on rVN-coated PVAI-C-20FGF and rVN-coated PVAI-C-80FGF hydrogels but could be cultured on rVN-coated CMC-C-5FGF dishes. These results indicate that not only the immobilization amount of FGF-2 but also base cell materials, including the rVN immobilization amount as well as the conformation and release of FGF-2 on the surface, should be important for human iPS cell cultivation.

The amount of FGF-2 immobilized on the rVN-coated CMC-C-5FGF and CMC-C-20FGF dishes was reduced to 93.6 and 52.2 times, respectively; thus, less FGF-2 was used compared to the conventional amount of FGF-2 used in culture medium for one passage (6 days) of human iPS cell culture. This reduction resulted from the stabilization of unstable FGF-2 by the immobilization of FGF-2, which was achieved using optimal base materials (CMC), coating materials (rVN) and long-joint segment (PEG4-SPDP) design.

### Characterization of human iPS cells after long-term culture on rVN-coated CMC-C-FGF dishes

**Figure 5.**
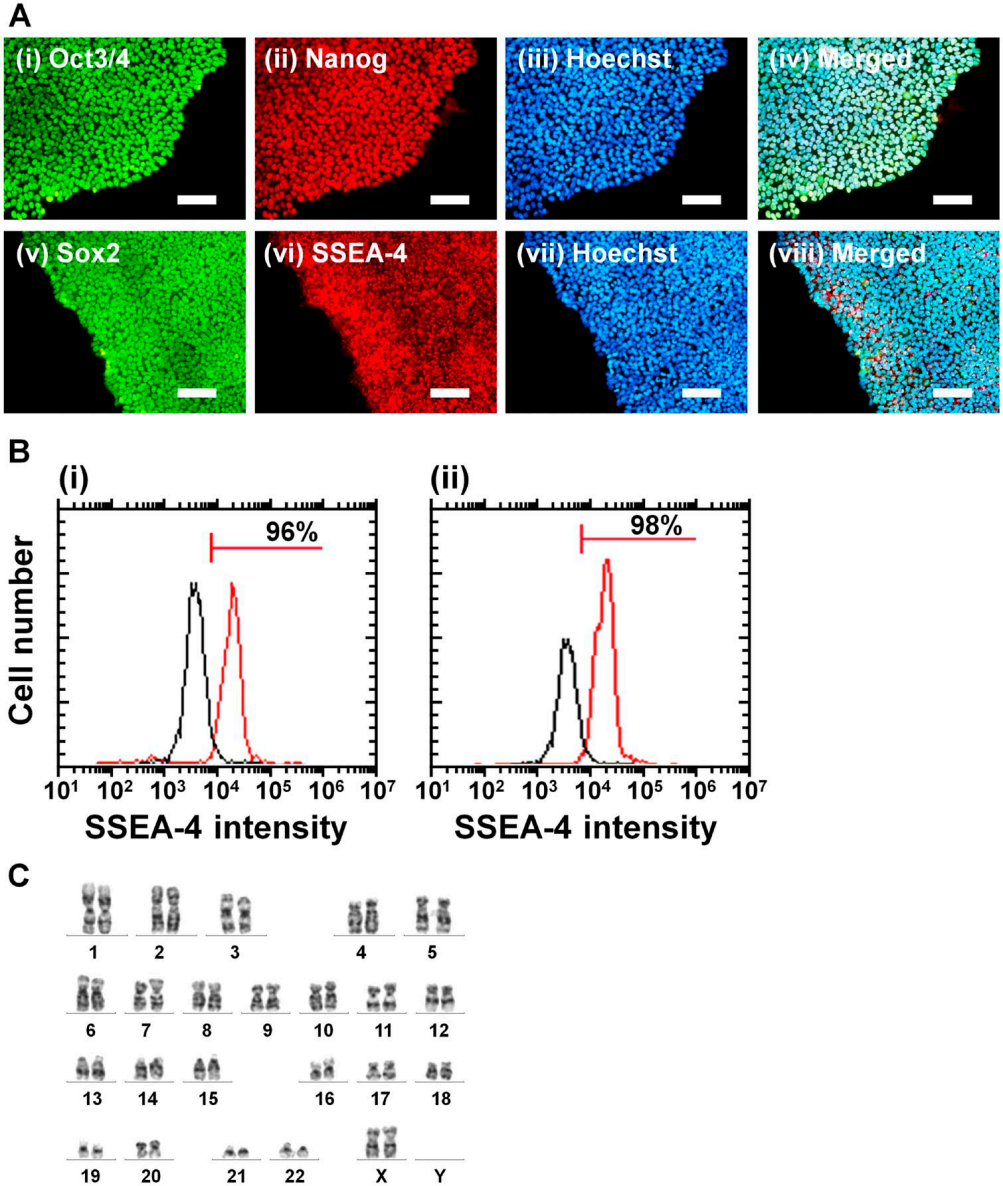
Pluripotency and differentiation potential of human iPS cells (HPS0077) *in vitro* and *in vivo* after long-term (10 passages) proliferation on rVN-coated CMC-C-20FGF dishes in E7 medium, which does not contain FGF-2, using xeno-free proliferation methods. (**A**) Expression of pluripotency protein of (**i**) Oct3/4, (**ii**) Nanog, (**v**) Sox2 and (**vi**) SSEA-4 in human iPS cells analysed utilizing immunostaining with nuclear staining by (**iii**, **vii**) Hoechst 33342 after human iPS cell culture on rVN-coated CMC-C-20FGF dishes for 10 passages in E7 medium. The images (**iv**) and (**viii**) were created by merging (**i**)–(**iii**) and (**v**)–(**vii**), respectively. The scale bar describes 100 μm. (**B**) SSEA-4 expression on hiPSCs (HPS0077), after proliferation on CMC-C-5FGF dishes (**i**) and rVN-coated CMC-C-20FGF dishes (**ii**) for 10 passages in E7 medium. (**C**) Karyotyping evaluation of human iPS cells after long-term (10 passages) proliferation on rVN-coated CMC-C-20FGF dishes for 10 passages in E7 medium.

We analysed whether human iPS cells could sustain their pluripotency and potential to induce differentiation into cells originated from three germ layers after long-term (10 passage) proliferation on rVN-coated CMC-C-FGF dishes in E7 medium. Therefore, we evaluated the expression of pluripotent proteins (Oct3/4, Nanog, Sox-2 and SSEA-4) on human iPS cells (HPS0077) after proliferation on CMC-C-5FGF and CMC-C-20FGF dishes for 10 passages in E7 medium (xeno-free condition) utilizing an immunohistochemical staining method, and the results are presented in Figure 5A and Supplementary Figure S3A, where Hoechst 33342 staining was used to find nuclei of human iPS cells. Human iPS cells displayed substantial expression of pluripotency proteins, even after long-term human iPS cell culture (10 passages) on CMC-C-20FGF dishes (Figure 5A) and CMC-C-5FGF dishes (Supplementary Figure S3A) in medium not containing FGF-2 (E7 medium).

The expression of pluripotent marker, SSEA-4, on human iPS cells (HPS0077) after proliferation on rVN-coated CMC-C-5FGF dishes and CMC-C-20FGF dishes for 10 passages in E7 medium was investigated utilizing flow cytometry, and the results are displayed in Figure 5B. The SSEA-4 expression on rVN-coated CMC-C-FGF dishes after long-term proliferation (10 passages), which was evaluated in this research, was higher than 95%, indicating that the differentiation ratio was equal or less than 5% from Equation (2).

Karyotype of human iPS cells (HPS0077) was evaluated after proliferation on rVN-coated CMC-C-20FGF dishes for 10 passages in E7 medium, and the results are displayed in Figure 5C. Karyotype of human iPS cells showed normal types, indicating that gene abnormality did not generate even after long-term proliferation (10 passages) of human iPS cells on rVN-coated CMC-C-20FGF dishes in E7 medium.

**Figure 6.**
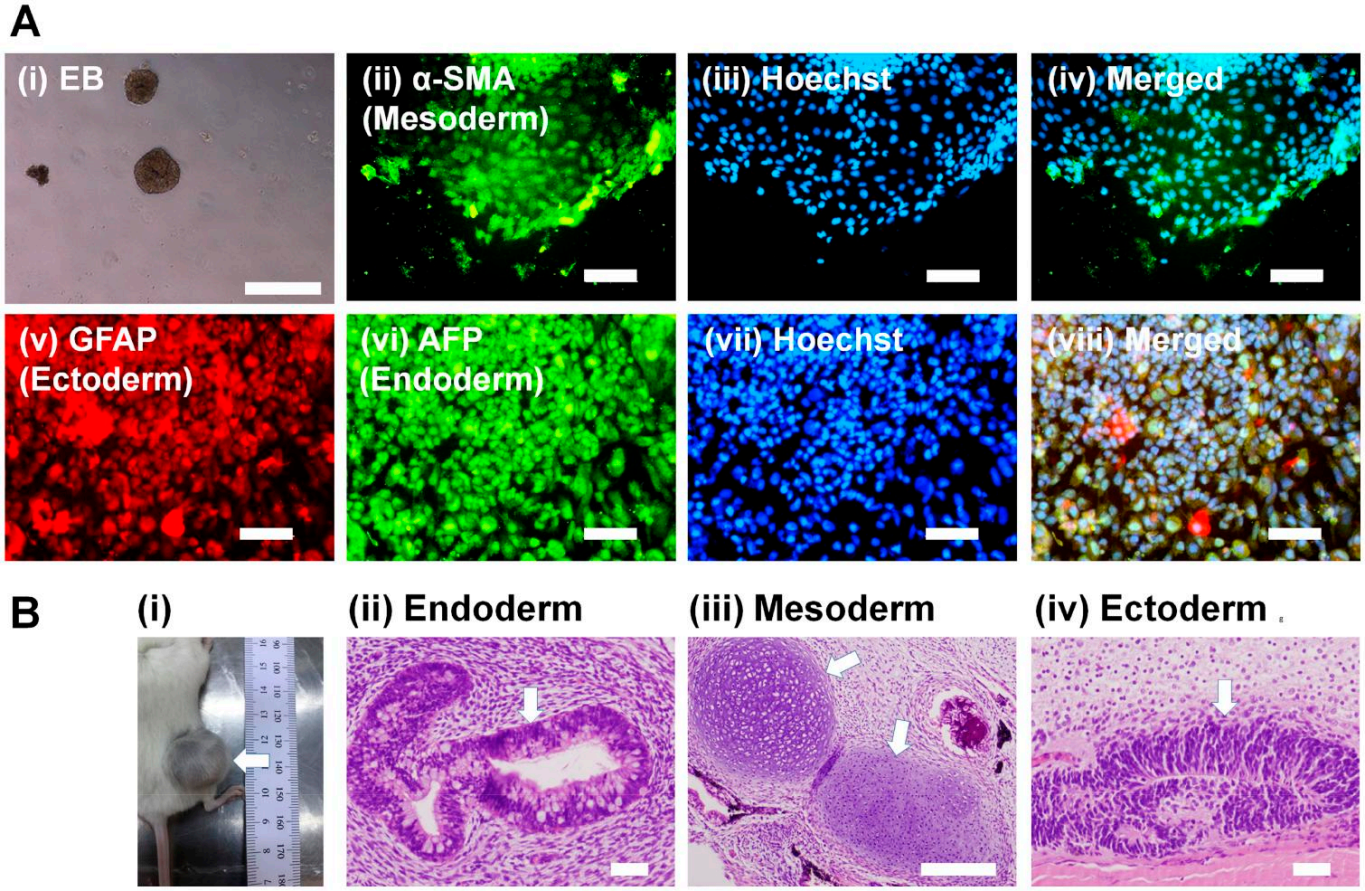
Differentiation potential of human iPS cells *in vitro* and *in vivo* after human iPS cell culture for 10 passages on rVN-coated CMC-C-20FGF dishes in E7 medium. (**A**) Differentiation potential of human iPS cells *in vitro* using an EB formation assay. (**i**) Morphologies of EB cells derived from differentiation of human iPS cells. (**ii**–**viii**) Expression of a mesodermal protein marker (**ii**, α-SMA), an ectodermal protein marker (**v**, GFAP) and an endodermal protein marker (**vi**, AFP) from EB cells analysed utilizing immunostaining with stained nuclears from Hoechst 33342 (**iii**, **vii** ) the pictures (**iv**) and (**viii**) were prepared by merging (**ii**)–(**iii**) and (**v**–**vii**), respectively. The scale bar is 500 μm (**i**) and 100 μm (**ii**–**viii**). (**B**) Differentiation potential of human iPS cells *in vivo* using a teratoma assay. (**i**) A teratoma generated by the transplantation of human iPS cells. (**ii**–**iv**) Tissues describing ducts made of columnar epithelium (**ii**, endoderm), cartilage (**iii**, mesoderm) and undifferentiated neuroepithelium (**iv**, ectoderm) were detected. The white arrows describe teratoma (**i**) and specific tissues (**ii**, **iii** and **iv**). The scale bar describes 100 μm (**ii**–**iv**).

It was also important to investigate whether human iPS cells could maintain their potential to induce differentiation into cells originating from three germ layers *in vivo* and *in vitro* to verify the pluripotent state of human iPS cells after their long-term cultivation on CMC-C-FGF dishes in media not including FGF-2. An EB formation analysis from human iPS cells (HPS0077) was proceeded to investigate the differentiation potential of human iPS cells *in vitro* after proliferation on CMC-C-5FGF and CMC-C-20FGF dishes for 10 passages in E7 medium under xeno-free methods. Figure 6A and Supplementary Figure S3B show the morphologies of EB formation and immunohistochemically analysed cells from EBs derived from three germ layers: (i) α-SMA-expressing cells, which indicate mesoderm cells, (ii) GFAP-expressing cells, which indicate ectoderm cells and (iii) AFP-expressing cells, which indicate endoderm cells, after human iPS cells were cultured on rVN-coated CMC-C-20FGF dishes (Figure 6A) and CMC-C-5FGF dishes (Supplementary Figure S3B) for 10 passages.

The cells derived from EBs substantially displayed marker proteins originating from the three germ layers, as depicted in Figure 6A and Supplementary Figure S3B, which indicated that human iPS cells sustained their potential to induce differentiated potential into cells originating from three germ layers *in vitro* even after proliferation on rVN-coated CMC-C-5FGF and CMC-C-20FGF in medium not containing FGF-2 (E7 medium) in xeno-free methods.

A teratoma generation assay was also performed to study the differentiation potential of human iPS cells into cells originating from three germ layers *in vivo*. After long-term (10 passages) proliferation of human iPS cells (HPS0077) on rVN-coated CMC-C-5FGF and CMC-C-20FGF dishes for 10 passages in E7 medium, which did not contain FGF-2 under xeno-free protocols, human iPS cells were subcutaneously injected into immune-deficient mice (NOD. CB17-*Prkdc*scid/Jnarl, NOD/SCID) to create teratomas (Figure 6Bi and Supplementary Figure S3Ci). The teratoma samples were proceeded to H&E staining after being fixed and sliced. Figure 6B and Supplementary Figure S3C show the cells originating from teratomas, which were created from human iPS cells (HPS0077) after long-term (10 passages) proliferation of the cells on rVN-coated CMC-C-20FGF (Figure 6B) and CMC-C-5FGF (Supplementary Figure S3C) dishes in E7 medium utilizing xeno-free methods. The cells originating from the endoderm layer (ducts made of columnar epithelium, Figure 6Bii and Supplementary Figure S3Cii), mesoderm layer (cartilage, Figure 6Biii and Supplementary Figure S3Ciii) and ectoderm layer (undifferentiated neuroepithelium, Figure 6Biv and Supplementary Figure S3Civ) were detected under each condition. Therefore, human iPS cells could differentiate into cells originating from three germ layers *in vivo*, even after proliferation on rVN-coated CMC-C-5FGF and CMC-C-20FGF dishes for 10 passages in E7 medium, which did not contain FGF-2, using xeno-free culture conditions.

### Cardiac differentiation of human iPS cells after long-term culture on rVN-coated CMC-C-FGF dishes

**Figure 7.**
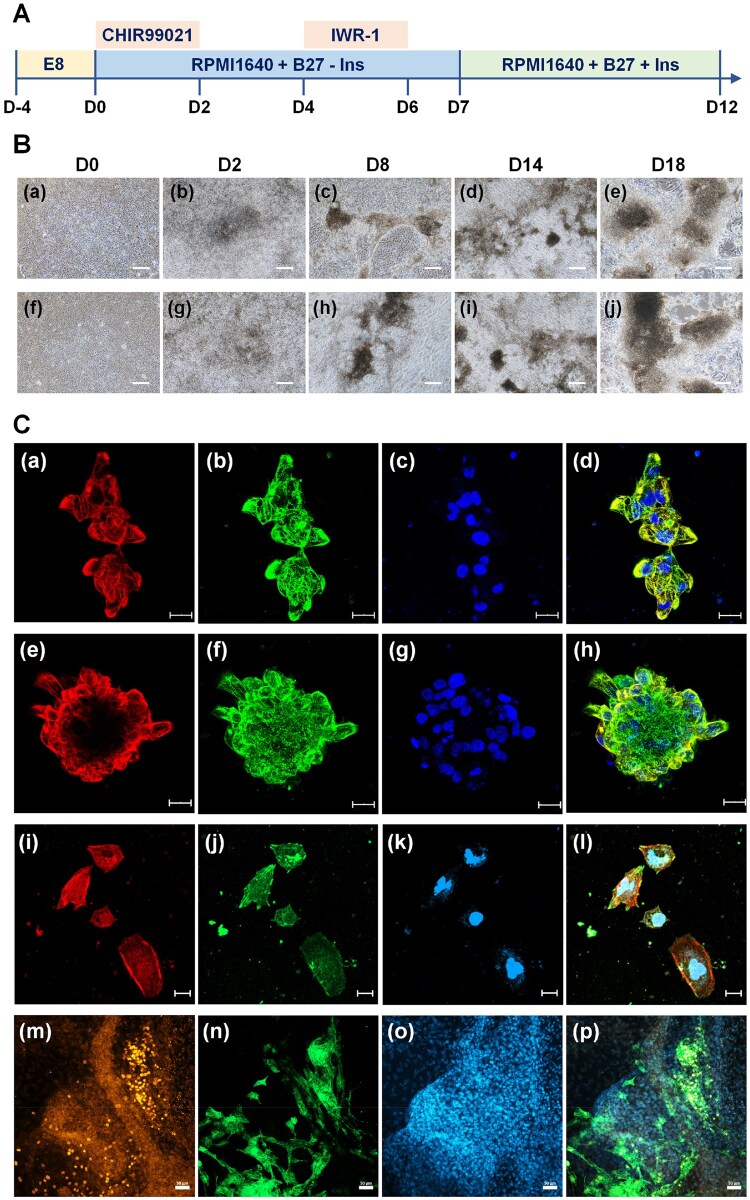
Cardiac differentiation of human iPS cells (HPS0077) after long-term (10 passages) proliferation on rVN-coated CMC-C-5FGF and CMC-C-20FGF dishes in E7 medium, which does not contain FGF-2, using xeno-free proliferation methods. (**A**) Timeline of the cardiac differentiation method of human iPS cells utilized in this work. (**B**) The sequential morphological observation during cardiac differentiation of human iPS cells on CMC-C-5FGF (**a**–**e**) and CMC-C-20FGF (**f**–**j**) dishes on day 0 (**a** and **f**), 2 (**b** and **g**), 8 (**c** and **h**), 14 (**d** and **i**) and 18 (**e** and **j**). Scale bar indicates 200 µm. (**C**) Immunohistochemical staining evaluation of human iPS cell-derived cardiac cells on CMC-C-5FGF (**a**–**d**, **i**–**l**) and CMC-C-20FGF (**e**–**h**, **m**–**p**) dishes. Expression of ML2CV (**a** and **e**), cTnT (**b** and **f**), α-actinin (**i**), cTnT (**j**) Nkx2.5 (**m**) and α-actinin (**n**) on human iPS cell-derived cardiac cells investigated by an immunohistochemical staining, which were differentiated on CMC-C-5FGF (**a**–**d**, **i**–**l**) and CMC-C-20FGF dishes (**e**–**h**, **m**–**p**) on day 18. DAPI (**c**, **g**, **k** and **o**, blue) was utilized for nuclei staining. The photos (**d**), (**h**), (**l**) and (**p**) were created by merging (**a**–**c**), (**e**–**g**), (**i**–**k**), and (**m**–**o**), respectively. The scale bar shows 20 µm (**a**–**l**) and 50 µm (**m**–**p**).

We evaluated whether human iPS cells (HPS0077) would differentiate into a specific type of cell, cardiac cells, after long-term (10 passages) cultivation on rVN-coated CMC-C-5FGF and CMC-C-20FGF dishes in E7 medium in xeno-free protocols. After 10 passages of culture on rVN-coated CMC-C-5FGF and CMC-C-20FGF dishes, human iPS cells were differentiated into cardiac cells utilizing a previously reported protocol (Figure 7A) [58]. The morphologies of human iPS cells during cardiac induction at days 0, 2, 8, 14 and 18 are shown in Figure 7B. The cells tended to aggregate with an enhanced time of cardiac induction. The beating cells appeared after 10 days of induction, where Supplementary Video S1 shows human iPS cell-derived cardiac cells on rVN-coated CMC-C-5FGF dishes and Supplementary Video S2 shows human iPS cell-derived cardiac cells on rVN-coated CMC-C-20FGF dishes. After 21 days of cardiac differentiation, we investigated the expression of the cardiac marker proteins of ML2Cv (mature ventricular cardiomyocytes), cTnT, α-actinin and Nkx2.5 on human iPS cell-differentiated cardiac cells (Figure 7C), which were cultivated on rVN-coated CMC-C-5FGF and CMC-C-20FGF dishes using immunostaining methods. In this study, the expression of cardiomyocyte markers, i.e. Nkx2.5, ML2Cv, α-actinin and cTnT, was extensively observed in human iPS cell-differentiated cardiac cells in this study.

These results suggested that human iPS cells proliferated on rVN-coated CMC-C-FGF dishes for 10 passages in E7 medium, which did not contain FGF-2, sustained their potential to induce to differentiation into targeted lineages of cells, such as cardiac cells, with good efficacy.

### Differentiation of human iPS cells into RPE cells after long-term proliferation on rVN-coated CMC-C-20FGF dishes

**Figure 8.**
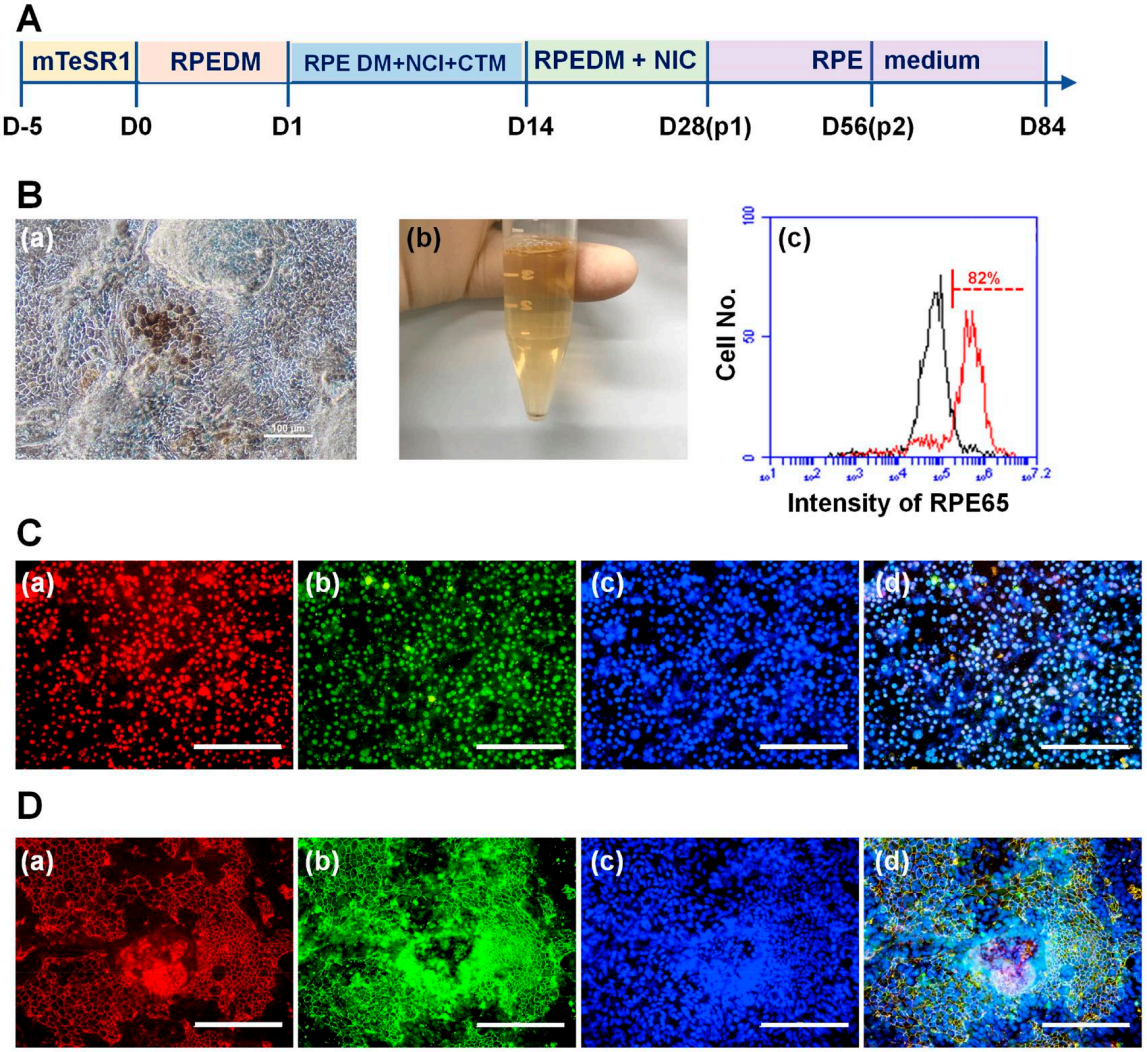
Differentiation of human iPS cells (HPS0077) into RPE cells after long-term (10 passages) proliferation on rVN-coated CMC-C-20FGF dishes in E7 medium, which does not contain FGF-2, using xeno-free proliferation methods. (**A**) Timeline of the RPE cell differentiation method of human iPS cells used in this work. (**B**) The morphological observation (**a**) and cell pellets (**b**) of human iPS cell-differentiated RPE cells after induction of 84 days. Scale bar shows 100 µm. Expression of RPE65 on human iPS cell-differentiated RPE cells analysed using flow cytometry after induction of 84 days (**c**). (**C**) Immunohistochemical staining evaluation of human iPS cell-differentiated RPE cells, after induction of 28 days. Expression of MITF (**a**) and PAX6 (**b**). DAPI (**c**) was utilized for nuclei staining. The photo (**d**) was created by merging (**a**–**c**). the scale bar shows 200 µm. (**D**) Immunohistochemical staining evaluation of human iPS cell-differentiated RPE cells, after induction of 86 days. Expression of ZO-1 (**a**) and RPE65 (**b**). DAPI (**c**) was utilized for nuclei staining. The photo (**d**) was created by merging (**a**–**c**). The scale bar shows 200 µm.

We evaluated whether human iPS cells (HPS0077) would differentiate into another specific type of cell, RPE cells, after long-term (10 passages) cultivation on rVN-coated CMC-C-20FGF dishes in E7 medium in xeno-free protocols. After 10 passages of culture on the rVN-coated CMC-C-20FGF dishes, human iPS cells were differentiated into RPE cells utilizing previously reported protocols [60, 61] with some modification (Figure 8A).

Pigmented cells (human iPS derived-RPE cells) started to find around day 44 of RPE induction and could be seen abundantly around day 56. After 84 days of differentiation, RPE cells showing polygonal cells were extensively observed (Figure 8Ba). The pigmented pellets could be isolated at day 84 (Figure 8Bb). The cells after differentiation into RPE cells for 84 days expressed 82% RPE marker of RPE65, which were analysed using flow cytometry (Figure 8Bc). We also analysed several RPE marker expression of human iPS cell-differentiated RPE cells using immunostaining method (Figure 8C and D) The differentiated cells expressed MITF (Figure 8Ca and d) and PAX6 (Figure 8Cb and d) markers after 28 days of induction into RPE cells. The differentiated cells also expressed ZO-1 (Figure 8Da and d) and RPE65 (Figure 8Db and d) markers after 84 days of induction into RPE cells.

These characterizations of human iPS cell-derived RPE cells confirmed the extensive differentiation of human iPS cells into mature RPE cells showing the expression of RPE-specific markers, even after human iPS cells proliferated on rVN-coated CMC-C-20FGF dishes for 10 passages in E7 medium.

## Conclusion

We developed two types of FGF-conjugated cell culture materials: rVN-coated PVAI-C-FGF hydrogels and rVN-coated CMC-C-FGF dishes. Three different conjugation amounts of FGF-2 were prepared on each material using 0.5, 2 and 8 μg/ml FGF-2 solution. Human iPS cells (HPS0077) could not be proliferated on rVN-coated PVAI-C-FGF hydrogels with any FGF-2 immobilization amount for over two passages in E7 medium, which did not contain FGF-2, whereas human iPS cells could be successfully cultivated on rVN-coated CMC-C-5FGF and CMC-C-20FGF dishes in E7 medium for 10 passages. These results suggest that not only the immobilization amount of FGF-2 but also the base cell materials, including the rVN immobilization amount as well as the conformation or release of FGF-2 on the surface, should be important. Human iPS cells cultured on rVN-coated CMC-C-FGF dishes in E7 medium for 10 passages sustained their pluripotency and differentiation potential into cells originating from three germ layers *in vitro* and *in vivo*. Furthermore, the cells could extensively differentiate into a specific cell lineage, cardiomyocytes and RPE cells. The FGF-2-immobilized surface could be useful for human PS cell culture because it allows the use of cell culture medium that does not contain unstable FGF-2.

## Supplementary Material

rbaf003_Supplementary_Data

## Data Availability

The data that support the findings of this study are available from the corresponding author upon reasonable request.
