# Correction to: Material surface conjugated with fibroblast growth factor-2 for pluripotent stem cell culture and differentiation

**DOI:** 10.1093/rb/rbag003

**Published:** 2026-01-29

Tzu-Cheng Sung, Zhi-Xian Pan, Ting Wang, Hui-Yu Lin, Chia-Lun Chang, Ling-Chun Hung, Suresh Kumar Subbiah, Remya Rajan Renuka, Shih-Jie Chou, Shih-Hwa Chiou, Idaszek Joanna, Henry Hsin-Chung Lee, Gwo-Jang Wu, Akon Higuchi, Material surface conjugated with fibroblast growth factor-2 for pluripotent stem cell culture and differentiation, *Regenerative Biomaterials*, Volume 12, 2025, rbaf003, https://doi.org/10.1093/rb/rbaf003

The following affiliation details have been revised to Taiwan, China:

2.) Department of Chemical and Materials Engineering, National Central University, Taoyuan 32001, Taiwan, China

4.) Department of Medical Research, Taipei Veterans General Hospital, Taipei 112201, Taiwan, China

5.) Institute of Pharmacology, School of Medicine, National Yang Ming Chiao Tung University, Taipei 112304, Taiwan, China

6.) Department of Ophthalmology, Taipei Veterans General Hospital, Taipei 11217, Taiwan, China

8.) Department of Surgery, Hsinchu Cathay General Hospital, Hsinchu 30060, Taiwan, China

9.) Graduate Institute of Translational and Interdisciplinary Medicine, National Central University, Taoyuan 32001, Taiwan, China

10.) Graduate Institute of Medical Sciences and Department of Obstetrics & Gynecology, Tri-Service General Hospital, National Defense Medical Center, Taipei 11490, Taiwan, China

11.) R&D Center for Membrane Technology, Chung Yuan Christian University, Taoyuan 320, Taiwan, China

