# Supplementary material for: Material surface conjugated with fibroblast growth factor-2 for pluripotent stem cell culture and differentiation
Source: Regen Biomater. 2025 Jan 2;12:rbaf003. doi: 10.1093/rb/rbaf003 (PMC11835233; doi:10.1093/rb/rbaf003)
Supplement: rbaf003_Supplementary_Data [file rbaf003_supplementary_data.zip › 36645_3_Supplementary(Regen_Biom)2024N2.pdf]

Material surface conjugated with fibroblast growth factor-2 for pluripotent stem cell culture and differentiation

Tzu-Cheng Sung[1,†], Zhi-Xian Pan[2,†], Ting Wang[1], Hui-Yu Lin[2], Chia-Lun Chang[2], Ling-Chun Hung[2], Suresh Kumar Subbiah[3], Remya Rajan Renuka[3], Shih-Jie Chou[4,5], Shih-Hwa Chiou[4-6], Idaszek Joanna[7], Henry Hsin-Chung Lee[8,9,***], Gwo-Jang Wu[10,**], Akon Higuchi[1,2,11,*]

[1] State Key Laboratory of Ophthalmology, Optometry and Visual Science, Eye Hospital, Wenzhou Medical University, No. 270, Xueyuan Road, Wenzhou, 325027, Zhejiang, China.

[2] Department of Chemical and Materials Engineering, National Central University, No. 300, Jhongda RD., Jhongli, 32001, Taoyuan, Taiwan, China.

[3] Center for Global Health Research, Saveetha Medical College and Hospitals, Saveetha Institute of Medical and Technical Sciences, Chennai, 602105, Tamil Nadu, India.

[4] Department of Medical Research, Taipei Veterans General Hospital, Taipei 112201, Taiwan, China.

[5] Institute of Pharmacology, School of Medicine, National Yang Ming Chiao Tung University, Taipei 112304, Taiwan, China.

[6] Department of Ophthalmology, Taipei Veterans General Hospital, Taipei 11217, Taiwan, China.

[7] Devision of of Materials Design, Faculty of Materials Science and Engineering, Warsaw University of Technology, 141 Woloska Street, Warsaw 02-507, Poland

[8] Department of Surgery, Hsinchu Cathay General Hospital, No. 678, Sec 2, Zhonghua Rd., Hsinchu, 30060, Taiwan, China.

[9] Graduate Institute of Translational and Interdisciplinary Medicine, National Central University, No. 300, Jhongda Rd., Jhongli, Taoyuan, 32001, Taiwan, China.

[10] Graduate Institute of Medical Sciences and Department of Obstetrics & Gynecology, Tri-Service General Hospital, National Defense Medical Center, Taipei, 11490, Taiwan, China.

[11] R&D Center for Membrane Technology, Chung Yuan Christian University, Jhongli, Taoyuan 320, Taiwan, China.

* Correspondence and requests for materials should be addressed to A.H. A.H. (e-mail: higuchi@ncu.edu.tw & higuchi@wmu.edu.cn), G.W. (e-mail: gwojang@yahoo.com), H.L. (e-mail: hsinchuoff@cgh.org.tw)

† These authors contributed equally.

Supplementary Information

Supplementary Table 1 Materials used in this study.

Materials	Abbreviation	Catalog No.	Company
		**ECM**	
Recombinant vitronectin	rVN	A14700	Thermo Fisher Scientific Inc. (Waltham, MA, USA)
		**Cell culture dishes**	
6-well polystyrene plate	TCP	#353046	Corning (Corning, NY, USA)
		**Chemicals & polymer**	
Poly(vinyl alcohol-co-itaconic acid)	PVAI	AF-17	Japan VAM & Poval Co. (Osaka, Japan)
O-carboxymethyl chitosan	CMC	sc-358091	Santa Cruz Biotechnology (Dallas, Texas, USA)
		**Cell culture medium and component**	
antibiotic-antimycotic	anti-anti	15240096	Thermo Fisher Scientific Inc. (Waltham, MA, USA)
chetomin	CTM	C9623	Sigma-Aldrich (St. Louis, MO, USA)
Essential 8 medium	Essential 8	A1517001	Thermo Fisher Scientific Inc. (Waltham, MA, USA)
DMEM/F12 medium	DMEM/F12 medium	11330-057	Thermo Fisher Scientific Inc. (Waltham, MA, USA)
Hoechst 33342	Hoechst	PA-3014	Lonza (Basel, Switzerland)
KnockOut Serum Replacement	KSR	10828028	Thermo Fisher Scientific Inc. (Waltham, MA, USA)
MEM nonessential amino acids	NEAA	11140050	Thermo Fisher Scientific Inc. (Waltham, MA, USA)
nicotinamide	NIC	N337621	Sigma-Aldrich (St. Louis, MO, USA)
RPMI 1640	RPMI 1640	11875093	Thermo Fisher Scientific Inc. (Waltham, MA, USA)
B-27™ Supplement, minus insulin	B27-	A1895601	Thermo Fisher Scientific Inc. (Waltham, MA, USA)
B-27™ Supplement	B27	17504044	Thermo Fisher Scientific Inc. (Waltham, MA, USA)
CHIR99021	CHIR99021	SML1046	Sigma-Aldrich (St. Louis, MO, USA)
IWR-1	IWR-1	I0161	Sigma-Aldrich (St. Louis, MO, USA)

Supplementary Table 1 (continued) Materials used in this study.

Materials	Abbreviation	Catalog No.	Company
		**Antibodies**	
Anti-Nanog (mouse IgG)	Anti-Nanog	MA1-017	Thermo Fisher Scientific Inc. (Waltham, MA, USA)
Anti-Oct3/4 (rabbit IgG)	Anti-Oct3/4	PA5-27438	Thermo Fisher Scientific Inc. (Waltham, MA, USA)
Anti-Sox2 (rabbit IgG)	Anti-Sox2	48-1400	Thermo Fisher Scientific Inc. (Waltham, MA, USA)
Anti-SSEA4 antibody (mouse IgG)	Anti-SSEA4 antibody	MA1-021	Thermo Fisher Scientific Inc. (Waltham, MA, USA)
Anti-α-fetoprotein (rabbit IgG)	Anti-AFP	PA5-21004	Thermo Fisher Scientific Inc. (Waltham, MA, USA)
Anti-glial fibrillary acidic protein (mouse IgG)	Anti-GFAP	MA5-15086	Thermo Fisher Scientific Inc. (Waltham, MA, USA)
Anti-smooth muscle actin (rabbit IgG)	Anti-SMA	PA5-19465	Thermo Fisher Scientific Inc. (Waltham, MA, USA)
Anti-ML2Cv antibody (rabbit IgG)	Anti-ML2Cv antibody	ab92721	Abcam (Milton, Cambridge, UK)
Anti-cTnT antibody (mouse IgG)	Anti-cTnT antibody	MA5-12960	Thermo Fisher Scientific Inc. (Waltham, MA, USA)
Anti-α-actinin antibody (mouse IgG)	Anti-α-actinin antibody	A7811	Sigma-Aldrich (St. Louis, MO, USA)
Anti-NKX2.5 antibody (rabbit IgG)	Anti-Nkx2.5 antibody	ab97355	Abcam (Milton, Cambridge, UK)
Anti-MITF (rabbit IgG)	Anti-MITF	MA5-32554	Thermo Fisher Scientific Inc. (Waltham, MA, USA)
Anti-PAX6 (mouse IgG)	Anti-PAX6	MA1-109	Thermo Fisher Scientific Inc. (Waltham, MA, USA)
Anti-RPE65 antibody (mouse IgG)	Anti-RPE65	MA1-16578	Thermo Fisher Scientific Inc. (Waltham, MA, USA)
Anti-ZO-1 (rabbit IgG)	Anti-ZO-1	600-401-GU7	Thermo Fisher Scientific Inc. (Waltham, MA, USA)
Mouse IgG1 Isotype antibody	Isotype-control	MA5-14453	Thermo Fisher Scientific Inc. (Waltham, MA, USA)
Alexa Fluor 488 goat anti-mouse IgG	Alexa Fluor 488 goat anti-mouse IgG	A-11001, A-11029	Thermo Fisher Scientific Inc. (Waltham, MA, USA)
Alexa Fluor 488 Goat Anti-Rabbit IgG	Alexa Fluor 488 goat anti-rabbit IgG	A11008	Thermo Fisher Scientific Inc. (Waltham, MA, USA)
Alexa Fluor 555 goat anti-rabbit IgG	Alexa Fluor 555 goat anti-rabbit IgG	A-21428	Thermo Fisher Scientific Inc. (Waltham, MA, USA)
Alexa Fluor 594 donkey anti-mouse IgG	Alexa Fluor 594 donkey anti-mouse IgG	A21203	Thermo Fisher Scientific Inc. (Waltham, MA, USA)

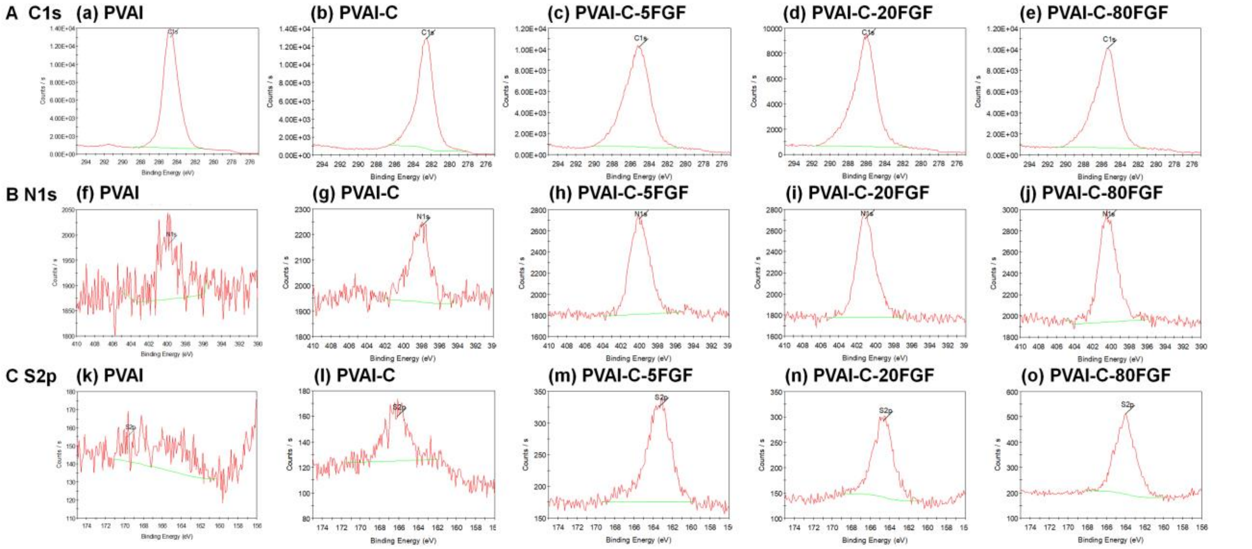

Supplementary Fig. 1 Surface analysis of PVAI hydrogels conjugated with FGF-2 by XPS. High-resolution spectra of the C 1s (A), N 1s (B), and S 2p (C) peaks of the surfaces of the PVAI (a, f, k), PVAI-C (b, g, l), PVAI-C-5FGF (c, h, m), PVAI-C-20FGF (d, i, n), and PVAI-C-80FGF (e, j, o) hydrogels.

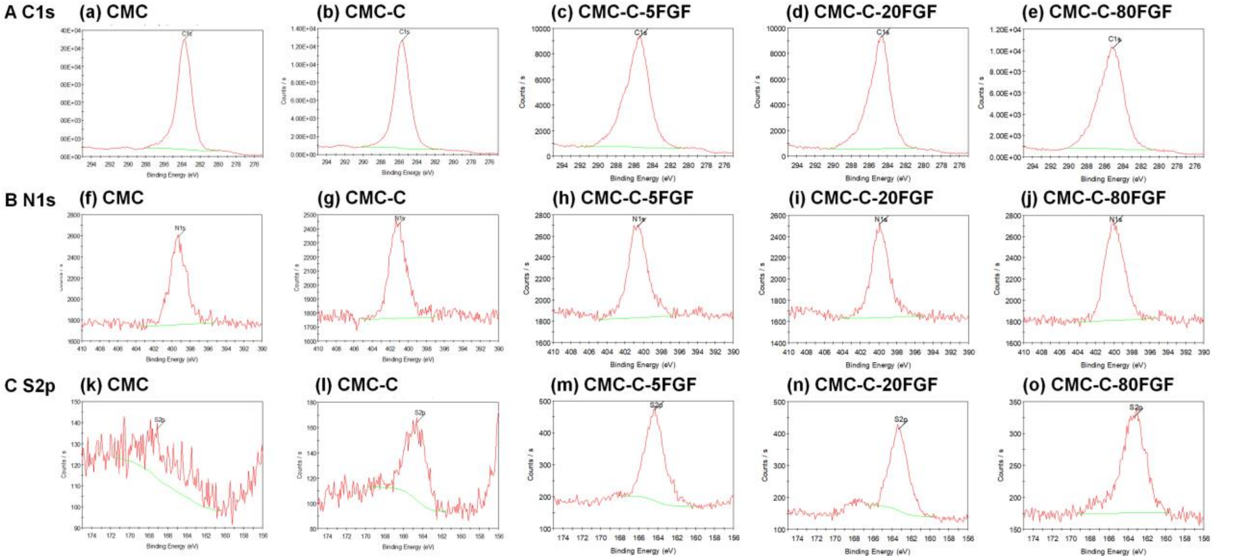

Supplementary Fig. 2. Surface analysis of CMC-coated dishes conjugated with FGF-2 by XPS. High-resolution spectra of the C 1s (A), N 1s (B), and S 2p (C) peaks of the surfaces of the CMC-coated dishes (a, f, k), CMC-C dishes (b, g, l), CMC-C-5FGF dishes (c, h, m), CMC-C-20FGF dishes (d, i, n), and CMC-C-80FGF dishes (e, j, o).

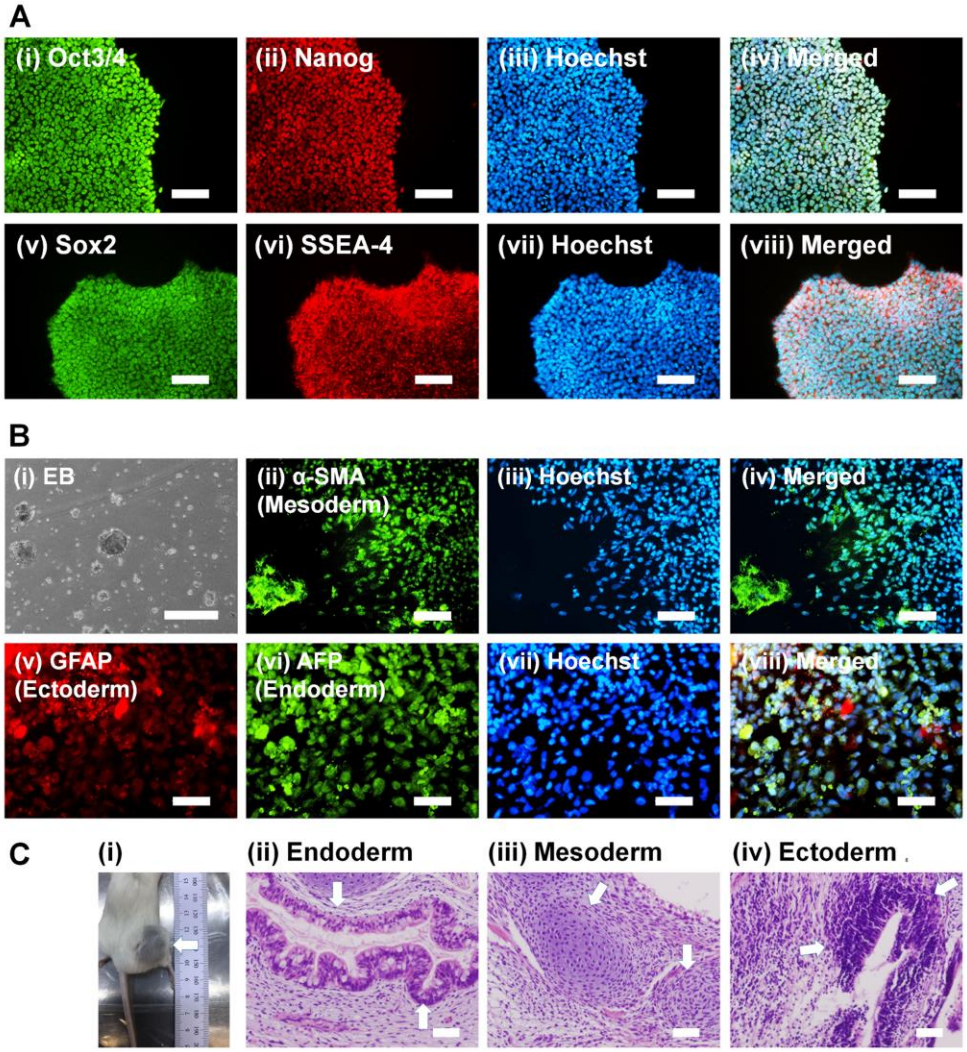

Supplementary Fig. 3. Pluripotency and differentiation ability of human iPS cells (HPS0077) *in vitro* and *in vivo* after long-term (passage ten) culture on rVN-coated CMC-C-5FGF dishes in E7 medium, which does not contain FGF-2, using xeno-free culture conditions. (A) Expression of pluripotency protein of Oct3/4 (i, green), Nanog (ii, red), Sox2 (v, green), and SSEA-4 (vi, red) in human iPS cells evaluated utilizing immunostaining with nuclear staining by Hoechst 33342 (blue, iii, vii) after human iPS cell culture on rVN-coated CMC-C-5FGF dishes for ten passages in E7 medium, which does not contain FGF-2, using xeno-free culture conditions. The images (iv) and (viii) were generated by merging (i)–(iii) and (v)–(vii), respectively. The scale bar is 100 μm. (B) Differentiation ability of human iPS cells *in vitro* using an EB formation assay after human iPS cell culture for ten passages on rVN-coated CMC-C-5FGF dishes in E7 medium, which does not contain FGF-2, using xeno-free culture conditions. (i) Morphologies of EB cells differentiated from human iPS cells. (ii-viii) Expression of a mesodermal marker protein (ii, α-SMA, green), an ectodermal marker protein (v, GFAP, red) and an endodermal marker protein (vi, AFP, green) from EB cells analyzed utilizing immunostaining with nuclear staining from Hoechst 33342 (iii, vii, blue) The photos (iv) and (viii) were created by merging (ii)–(iii) and (v)–(vii), respectively. The scale bar is 500 μm (i) and 100 μm (ii)-(viii). (C) Differentiation ability of human iPS cells *in vivo* using a teratoma assay after human iPS cell culture for ten passages on rVN-coated CMC-C-5FGF dishes in E7 medium, which does not contain FGF-2, using xeno-free culture conditions. (i) A teratoma generated by the transplantation of human iPS cells. (ii-iv) Tissues including ducts composed of columnar epithelium (ii, endoderm), cartilage (iii, mesoderm) and undifferentiated neuroepithelium (iv, ectoderm) were detected. The white arrows indicate teratoma (i) and specific tissues (ii, iii and iv). The scale bar is 100 μm (ii-iv).
